# Diversity and community composition of strictly anaerobic and culturable bacteria from the feces of Styrofoam-fed *Tenebrio molitor* larvae: a culturomics-based study

**DOI:** 10.3389/fmicb.2023.1309806

**Published:** 2023-12-05

**Authors:** Junyu Zhu, Xiaochen Chen, Sheng-Chung Chen, Wanling Qiu, Jianying Yu, Tengfei Guo, Xianxing Wang

**Affiliations:** ^1^Innovation Center for Soil Remediation and Restoration Technologies, College of Environment and Safety Engineering, Fuzhou University, Fuzhou, Fujian, China; ^2^School of Resources and Chemical Engineering, Sanming University, Sanming, Fujian, China; ^3^The Second Geological Exploration Institute, China Metallurgical Geology Bureau, Fuzhou, Fujian, China

**Keywords:** *Tenebrio molitor*, gut microbiome, feces, anaerobic bacteria, bacterial diversity, Styrofoam, polystyrene

## Abstract

**Introduction:**

In recent years, researchers have been exploring the plastic-degrading abilities of bacteria residing in the guts of Styrofoam-eating *Tenebrio molitor* larvae. However, none of the reported strains have displayed highly efficient plastic degradation capabilities, and it’s noteworthy that none of the existing studies have focused on strictly anaerobic microbes.

**Methods:**

In this study, we exclusively fed Styrofoam to *T. molitor* larvae and examined how this dietary change influence the gut’s bacterial community composition, as observed through fecal bacteria using bacterial 16S rRNA gene amplicon sequencing and the small-scale culturomics method with 20 types of anaerobic media under four different conditions.

**Results:**

The results revealed a significant shift in the dominant phylogroup from *Lactococcus* (37.8%) to *Escherichia–Shigella* (54.7%) when comparing the feces of larvae fed with bran and Styrofoam, as analyzing through the bacterial 16S rRNA gene amplicon sequencing. For small-scale culturomics method, a total of 226 strains of anaerobic bacteria were isolated and purified using the rolling-tube/strictly anaerobic technique. Among them, 226 strains were classified into 3 phyla, 7 classes, 9 orders, 17 families, 29 genera, 42 known species and 34 potential novel species.

**Discussion:**

Interestingly, 24 genera in total, identified through the culturomics method, were not found in the results obtained from amplicon sequencing. Here, we present a collection of culturable anaerobic bacteria from the feces of *T. molitor* larvae, which might be a promising avenue for investigating the biodegradability of plastics by combining specific strains, either randomly or intentionally, while considering the abundance ratio of the microbial community composition.

## 1 Introduction

The extensive utilization of plastic products, combined with inadequate handling and disposal practices, has led to a substantial buildup of plastic waste. This issue not only contributes to environmental contamination and destruction of plant habitats but also represents a somewhat inefficient use of resources (Geyer et al., 2017; Chamas et al., 2020; Lange, 2021). Conventional methods for managing plastic waste, such as landfills and incineration, conceal latent hazards and inflict harm upon the environment. These approaches are currently encountering limitations and are incapable of adequately combating plastic pollution (Zhang et al., 2021).

Bioremediation is frequently considered a more environmentally friendly and eco-conscious treatment approach. Furthermore, the emergence of plastic-eating insects, such as *Tenebrio molitor*, *Zophobas morio*, and *Galleria mellonella*, offers a novel strategy for addressing plastic pollution (Kim et al., 2020; Brandon et al., 2021; Wang et al., 2022). During the consumption process, the gut microbiota plays a crucial role, participating in and regulating the development of the host’s immune system and the process of nutrient metabolism while also providing feedback on the host’s physiological status (Sekirov et al., 2010). In recent years, numerous researchers have attempted to isolate and culture bacteria with efficient plastic biodegradability within the digestive tracts of these insects (Lee et al., 2020; Lou et al., 2020, 2021). However, none of the reported strains thus far have demonstrated highly efficient plastic degradation capabilities (Urbanek et al., 2020; Zhang et al., 2022).

The rise of metagenomics has unveiled the diversity of the gut microbiota, yet it has also illuminated that the majority of gut microbiota remain uncultured or exist in extremely low abundance (Hugenholtz and Tyson, 2008). In fact, it was previously estimated that for environmental bacteria, only 1% can be cultivated using conventional techniques (Hofer, 2018). This may explain why we have not yet been able to isolate efficient plastic-degrading bacteria from the digestive tracts of these plastic-eating insects. The resurgence of culture techniques in microbiology, known as culturomics, was developed to culture and identify previously unknown microbiota residing in the gut (Lagier et al., 2016). In essence, the culturomics approach involves employing multiple media methods combined with different culture conditions to significantly increase the variety and quantity of culturable strains. This not only validates the metagenomic finding but also constitutes an essential step toward successfully uncovering how gut microbes maintain an organism’s homeostatic balance of gut microecosystems after exposure to pollutants.

In addition, it is worth noting that none of the current studies have discussed strictly anaerobic microbes, despite the fact that anaerobic flora constitutes a significant portion of the gut microbiota. So our study here was to alter the gut microecology of *T. molitor* larvae by feeding them with Styrofoam. Meanwhile, *T. molitor’s* fecal samples were high-throughput sequenced to reflect their gut microbial community composition, as such microbiota information can better reflect the host’s dietary status. Moreover, we used a culturomics approach to investigate the diversity of strictly anaerobic and culturable bacteria in the feces of Styrofoam-fed *T. molitor.* We hypothesized that changes in diet structure would impact gut microecology, and utilizing a variety of culture methods and conditions would significantly expand the types and quantities of culturable bacteria. We aim for this pioneering research to fill the existing gaps in the study of anaerobes in the gut of *T. molitor* larvae, offering a potential approach to broaden the avenues of plastic biodegradation and ultimately contribute to addressing the environmental problems arising from the accumulation of plastic waste.

## 2 Materials and methods

### 2.1 Source of *Tenebrio molitor* larvae and their feces collection

*Tenebrio molitor* larvae were purchased in November 2020 from “Pan Nongjia Manor,” an online store that specialized in selling mealworms in Shaoxing, Zhejiang Province, China. One batch of larvae was exclusively fed Styrofoam and kept indoors in a plastic container with a transparent lid at room temperature (~25°C). After feeding the *T. molitor* larvae using this method for over 6 months, fresh feces were collected daily with a sterile spoon and placed into sterilized microcentrifuge tubes, which were then stored at 4°C for subsequent experiments. Another batch of larvae fed bran as the control group.

### 2.2 Gut bacterial diversity analysis using full-length 16S rRNA gene amplicon sequencing

The feces collected from *T. molitor* larvae fed bran (control group, CG) or Styrofoam (treatment group, TG) were sent to Guangdong Magigene Technology Co., Ltd. and Genomics BioSci & Tech Co., Ltd., respectively. The near-full-length 16S rRNA gene (V1–V9) amplicon sequencing was performed using the PacBio Sequel IIe sequencer with the primer sets 27F (5’-AGRGTTYGATYMTGGCTCAG-3′) and 1492R (5’-RGYTACCTTGTTACGACTT-3′) (Lane et al., 1985; Weisburg et al., 1991; Vergin et al., 1998). The pooled 16S amplicons (600 ng) were used as templates to prepare the SMRTbell library via the SMRTbell Express template prep kit 2.0 (PacBio, Menlo Park, CA, USA), according to the manufacturer’s instructions. After steps for damage repair and end repair, the inserts were ligated to adapters. The SMRT sequencing was performed on an SMRT 8 M cell (PacBio, Menlo Park, CA, USA) with chemistry version 2 on the PacBio Sequel IIe sequencer. A primary filtering analysis was achieved on the Sequel IIe System, and the secondary analysis was completed using the SMRT analysis pipeline version 11.0.0. The subreads were transferred into circular consensus sequencing (CCS) reads with at least 0.99 accuracy and 3 passes. Then, the CCS reads were de-multiplexed by the SMRTLink analysis pipeline version 9.0. Sequences from both ends of the 27F - 1492R primers were trimmed by Cutadapt (version 1.16) (Martin, 2011) with the following criteria: a minimum length of 600 bps. Chimeric sequences were detected and discarded by the UCHIME algorithm with reference to the Gold database (Edgar et al., 2011; Haas et al., 2011). The filtered sequences were clustered into operational taxonomic units (OTUs) at 97% identity by Mothur (version 1.44.0) software with the SILVA database (SILVA 132) (Ludwig et al., 2004; Schloss et al., 2009; Quast et al., 2013; Yilmaz et al., 2014; Glockner et al., 2017) and further analyzed by QIIME (version 1.9.0) (Caporaso et al., 2010).

### 2.3 Anaerobic media selection and culture conditions

Methanogens, or methanogenic archaea, are strictly anaerobic microorganisms that inhabit all types of anoxic niches (Lyu et al., 2020; Thomas et al., 2022), forming syntrophic partnerships with diverse fermentative bacteria (Sieber et al., 2012), and playing a significant role in the global carbon cycle (Conrad, 2020). Thus, methanogens’ media appear to be a good choice for isolating diverse anaerobes in anoxic habitats. At Leibniz Institute DSMZ-German Collection of Microorganisms and Cell Cultures, one of the largest biological resource centers worldwide, a total of 39 types of media were used for the activation and preservation of methanogens, as listed in Supplementary Table S1. Out of them, 20 were selected (as shown in Supplementary Table S1) for the small-scale culturomics strategy employed to explore and isolate diverse anaerobes in the gut of *T. molitor* in this study. All anaerobic media were prepared under an oxygen-free N_2_/CO_2_ (80:20) atmosphere, according to our previous studies (Lai et al., 2002; Weng et al., 2015).

To isolate diverse *T. molitor* larvae gut anaerobes, two factors were taken into consideration: carbon source and salinity. First, acetate, formate, methanol, and trimethylamine (abbreviated as AFMT), which are known metabolic substrates for various methanogens, are used as carbon sources to culture methanogens and the anaerobic microbes that co-exist with them (Enzmann et al., 2018; Kurth et al., 2020). Second, AFMT was replaced with 1% PS particles (~300 μm, white amorphous particles, purchased from “Mingshuo Chemical,” an online store that specialized in selling plastic powder in Dongguan, Guangdong Province, China), which is expected to be utilized as an additional carbon source by strains that can biodegrade PS (Urbanek et al., 2020). Finally, the culture conditions include low salt (0% NaCl) and high salt (3% NaCl) to stimulate a water-deficient environment, as *T. molitor* feces, also known as fecal sand, are naturally almost water-free (Jin-hua, 2012). These conditions are included in detail: (1) 0% NaCl +1% PS particles; (2) 3% NaCl +1% PS particles; (3) 0% NaCl +25 mM AFMT; and (4) 3% NaCl + AFMT. All cultures were incubated at room temperature (~25°C).

### 2.4 Inoculation, isolation, and purification of *Tenebrio molitor* larvae gut anaerobes

**Figure 1:**
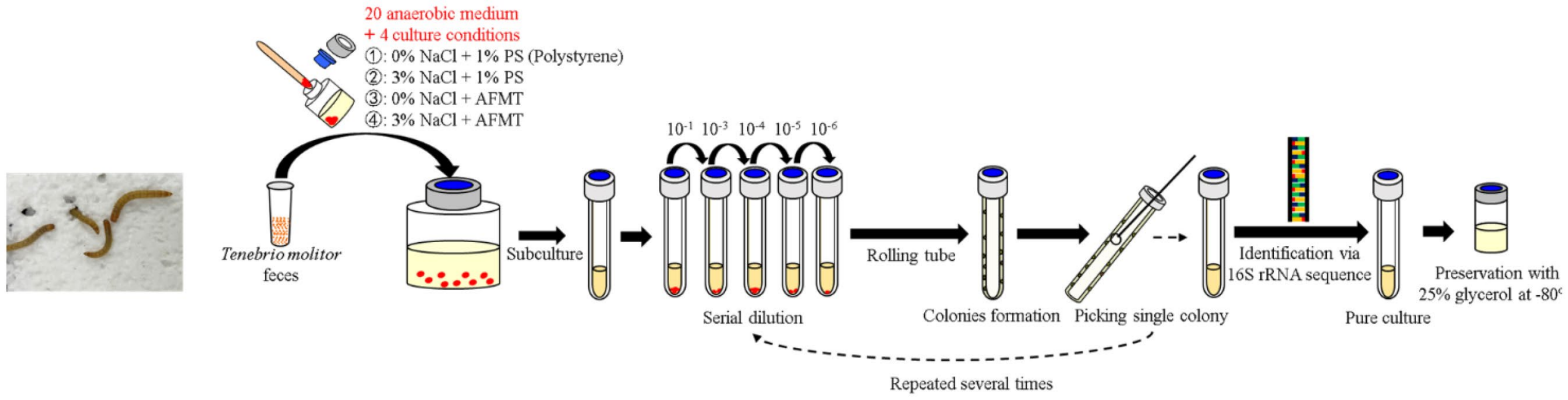
Flowchart of inoculation, enrichment, isolation, purification, and preservation of gut anaerobes of *T. molitor* larvae.

The collected feces were inoculated into 20 types of anaerobic media with four different conditions in May 2021 and incubated at room temperature (~25°C) for 3–4 months. Then the rolling-tube technique (Hungate, 1969) was performed in September 2021 for each condition of media. After colony formation, different types of colonies were selected and picked into fresh liquid media under their original conditions in the anaerobic grove box (UNIlab Pro Sp, MBraun, Germany). All isolated strains were preserved in their own media, contained 25% glycerol at –80°C. For further purification, the isolated strains were passed through a rolling-tube and picking colony for 2–3 rounds until they were pure. Meanwhile, 16S rRNA gene clone sequencing was performed to identify these isolated strains (Figure 1).

### 2.5 Identification of purified isolates using 16S rRNA gene clone sequencing

The 16S rRNA gene fragment of each purified isolate was amplified using bacterial 16S rRNA gene universal primer set of 8F (5’-AGAGTTTGATCCTGGCTCAG-3′) (Gray and Herwig, 1996) and 1492RU (5’-TTTTAATTAAGGTTACCTTGTTACGACTT-3′) (Turner et al., 1999), which were ordered from Sangon Biotechnology (Shanghai) Co., Ltd. The PCR cycling parameters were set as follows: initial denaturation at 94°C for 5 min; 2 cycles at 94°C 30 s, 56°C 30 s, 72°C 90 s; 2 cycles at 94°C 30 s, 54°C 30 s, 72°C 90 s; 2 cycles at 94°C 30 s, 52°C 30 s, 72°C 90 s; 30 cycles at 94°C 30 s, 50°C 30 s, 72°C 90 s; 72°C for 10 min; final step kept at 4°C. The verified PCR amplification products (~1500 bp) were then sent to Sangon Biotechnology (Shanghai) Co., Ltd. for clone sequencing. Bacterial identification of obtained sequences was performed at the websites of EzBioCloud’s identification service^1^ (Yoon et al., 2017) and NCBI^2^ with Nucleotide BLAST for searching the rRNA database (McGinnis and Madden, 2004). If the similarity between the query 16S rRNA gene of the purified strain and the most closely related strain or sequence is greater than 98.65%, the purified isolate is considered to belong to the same species as the closest strain. If the similarity is less than 98.65% but greater than 95.0%, the strain is considered a novel species (Kim et al., 2014). Additionally, if the similarity is less than 95.0%, the isolate is classified as a novel genus (Van Trappen et al., 2003).

### 2.6 Phylogenetic analysis of 16S rRNA gene sequences

In addition to the 16S rRNA gene sequences obtained from 226 strains in this study, their related 16S rRNA gene sequences were retrieved from the NCBI Reference Sequence Database and the GenBank Database. Phylogenetic trees were reconstructed using the MEGA11 program (Tamura et al., 2021) with the neighbor-joining method, employing 1,000 bootstrap replicates.

## 3 Results

### 3.1 Dominant and differentially abundant gut bacterial communities in *Tenebrio molitor* larvae fed with bran (CG) and Styrofoam (TG)

**Figure 2:**
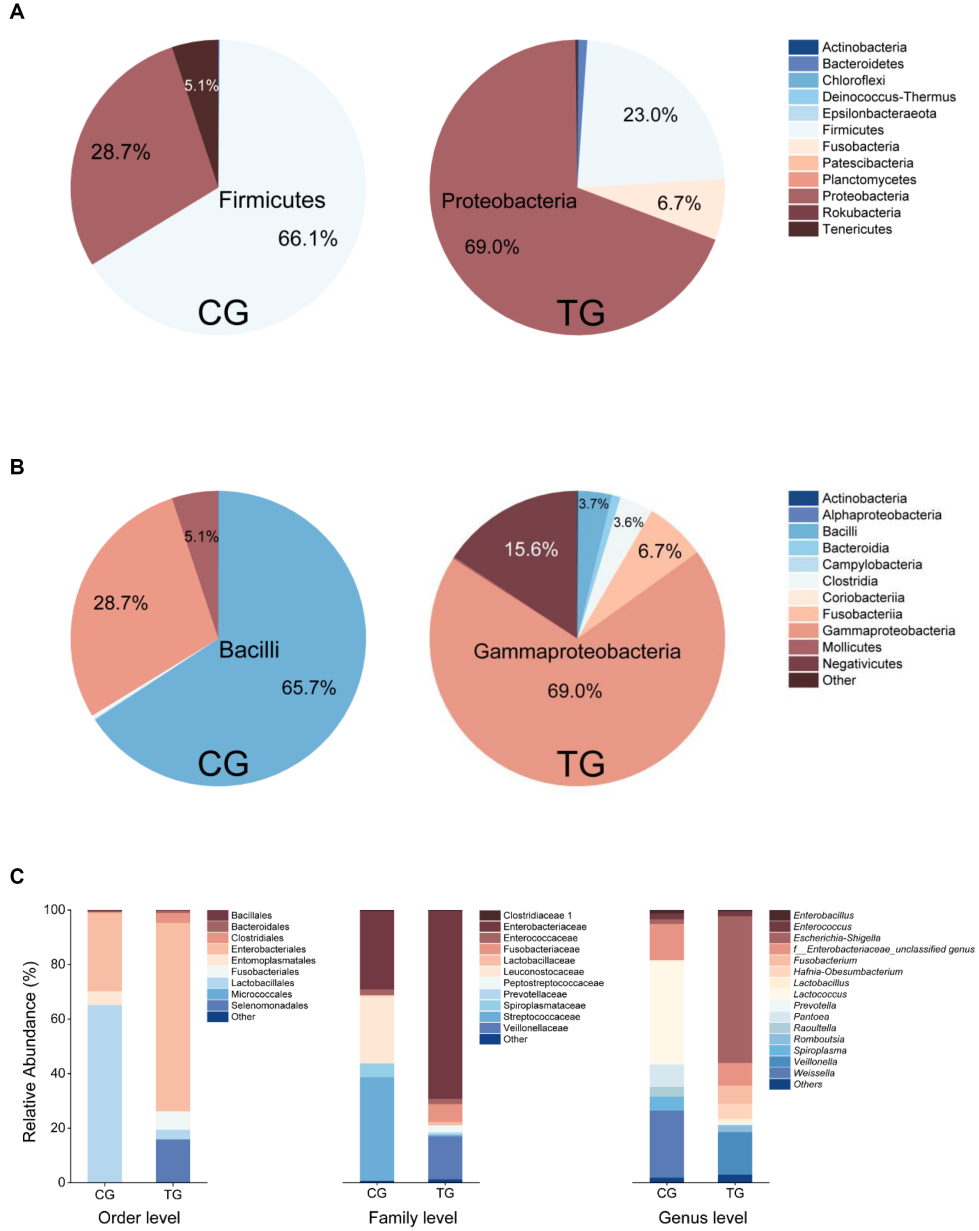
Fecal bacterial community composition in the control group (CG, fed bran) and treatment group (TG, fed Styrofoam) at the phylum level **(A)**, class level **(B)**, and different taxonomic levels **(C)**.

Based on the analyses of bacterial 16S rRNA gene amplicon sequencing, the most relatively abundant phyla in the control group (CG) were Firmicutes (66.1%), Proteobacteria (28.7%), and Tenericutes (5.1%). In the treatment group (TG), Proteobacteria (69.0%), Firmicutes (23.0%), and Fusobacteria (6.7%) were dominant (Figure 2A). At the class level, the more relatively abundant classes were Bacilli (65.7%), Gammaproteobacteria (28.7%), and Mollicutes (5.1%) in the CG. In the TG, Gammaproteobacteria (69.0%) was the most dominant class, and then the more abundant classes were Negativicutes (15.6%) and Fusobacteriia (6.7%) (Figure 2B). At the genus level, the dominant genera were *Lactococcus* (37.8%), *Weissella* (24.7%), *Pantoea* (8.3%), and *Spiroplasma* (5.1%) in the CG. For the TG, the most dominant phylogroup is *Escherichia–Shigella* with the highest abundance of 54.7%. Other dominant genera were *Veillonella* (15.7%), f_Enterobacteriaceae_unclassified genus (8.3%), *Fusobacterium* (6.6%), and *Hafnia-Obesumbacterium* (5.7%) in the TG (Figure 2C). The microbial community composition of the TG was significantly different from that of the CG, suggesting that feeding Styrofoam significantly altered the gut microbial community structure of *T. molitor* larvae (Figure 2).

### 3.2 Diversity of strictly anaerobic and culturable bacteria isolated from the feces of *Tenebrio molitor* larvae fed Styrofoam

A total of 226 strains of strictly anaerobic bacteria were isolated and purified using 20 different types of media under four different conditions (Figure 1). Through 16S rRNA gene clone sequencing and phylogenetic analysis, these 226 strains were classified into 3 phyla, 7 classes, 9 orders, 17 families, and 29 genera (Supplementary Table S2 and Supplementary Figures S1–S9). Additionally, 192 of these strains shared a similarity of ≧98.65% with known species, while the remaining 34 strains could potentially represent novel species, as they share less than 98.65% similarity in 16S rRNA gene sequences with known species, including 11 strains that could potentially belong to novel genera (Supplementary Table S2).

**Figure 3:**
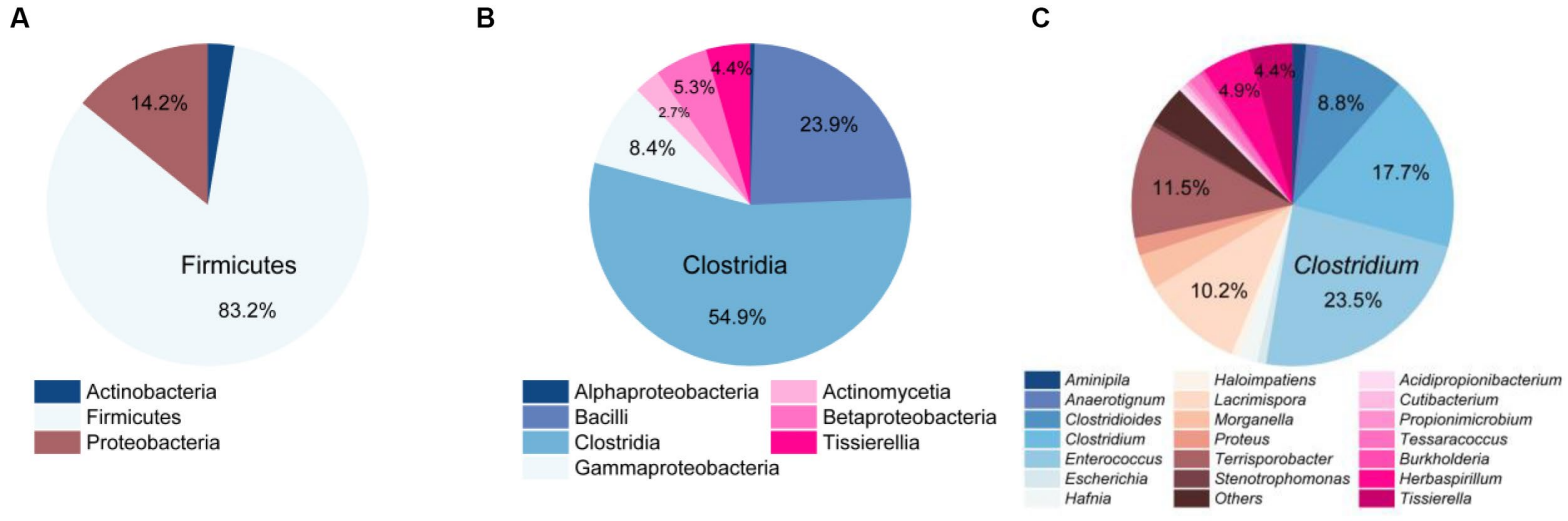
Community composition of strictly anaerobic and culturable bacteria in this study. At the phylum level **(A)**, class level **(B)**, and genus level **(C)**.

At the phylum level, Firmicutes was the most dominant group with 188 strains, accounting for 83.2% of the total. It was followed by Proteobacteria with 32 strains, representing 14.2%, and Actinobacteria with 6 strains, making up 2.7% (Figure 3A). At the class level, Clostridia (124 strains) and Bacilli (54 strains) were the predominant classes, constituting 54.9 and 23.9% of the total bacterial population, as illustrated in Figure 3B. At the genus level, the top five abundant genera were *Enterococcus* (23.5%), *Clostridium* (17.7%), *Terrisporobacter* (11.5%), *Lacrimispora* (10.2%), and *Clostridioides* (8.8%) (Figure 3C).

**Figure 4:**
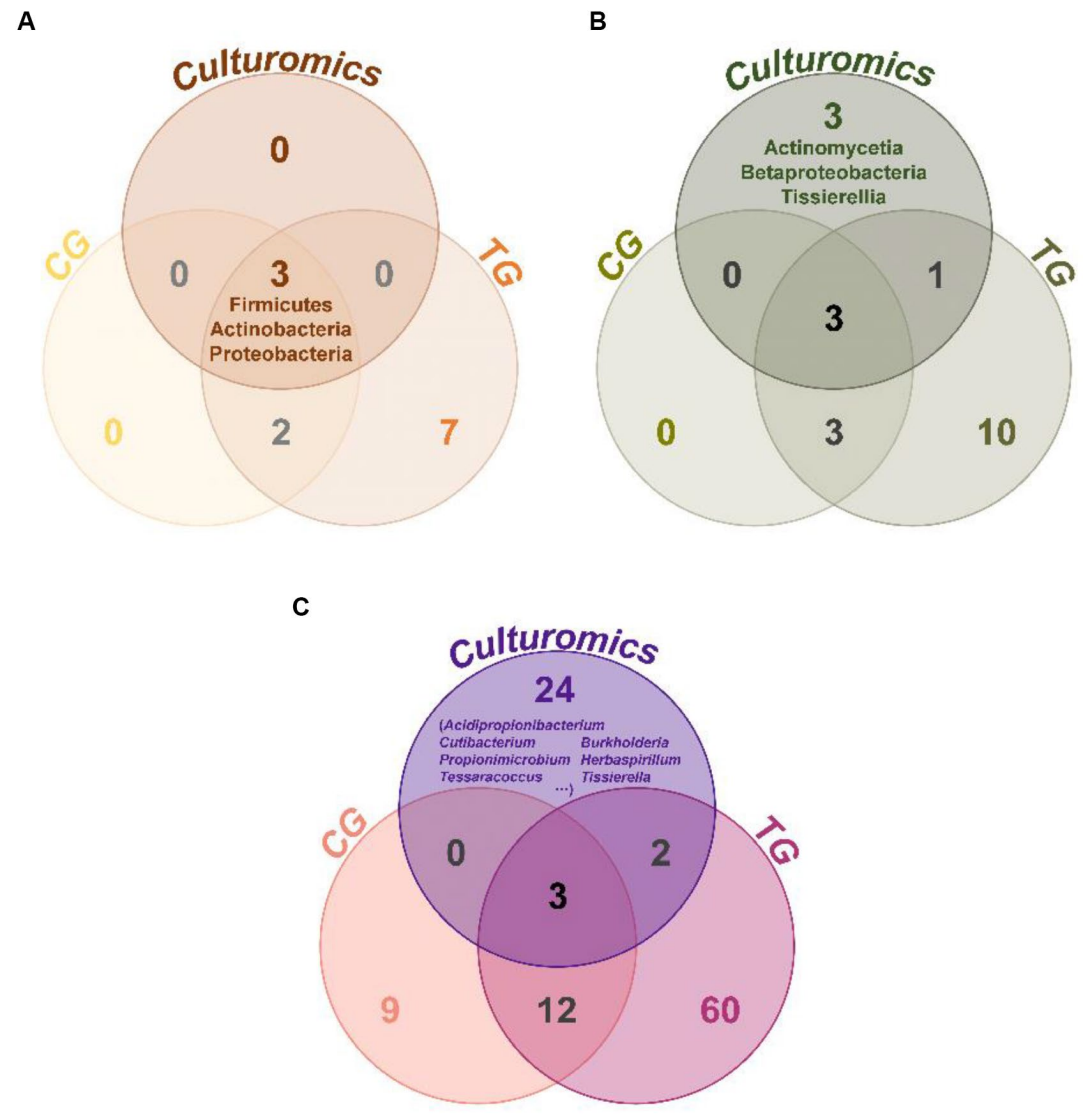
Venn diagram of taxonomic group analysis between the *T. molitor*’s feces isolate culturable bacteria (culturomics), control group (CG), and treatment group (TG). At the phylum level **(A)**, class level **(B)**, and genus level **(C)**.

**Figure 5:**
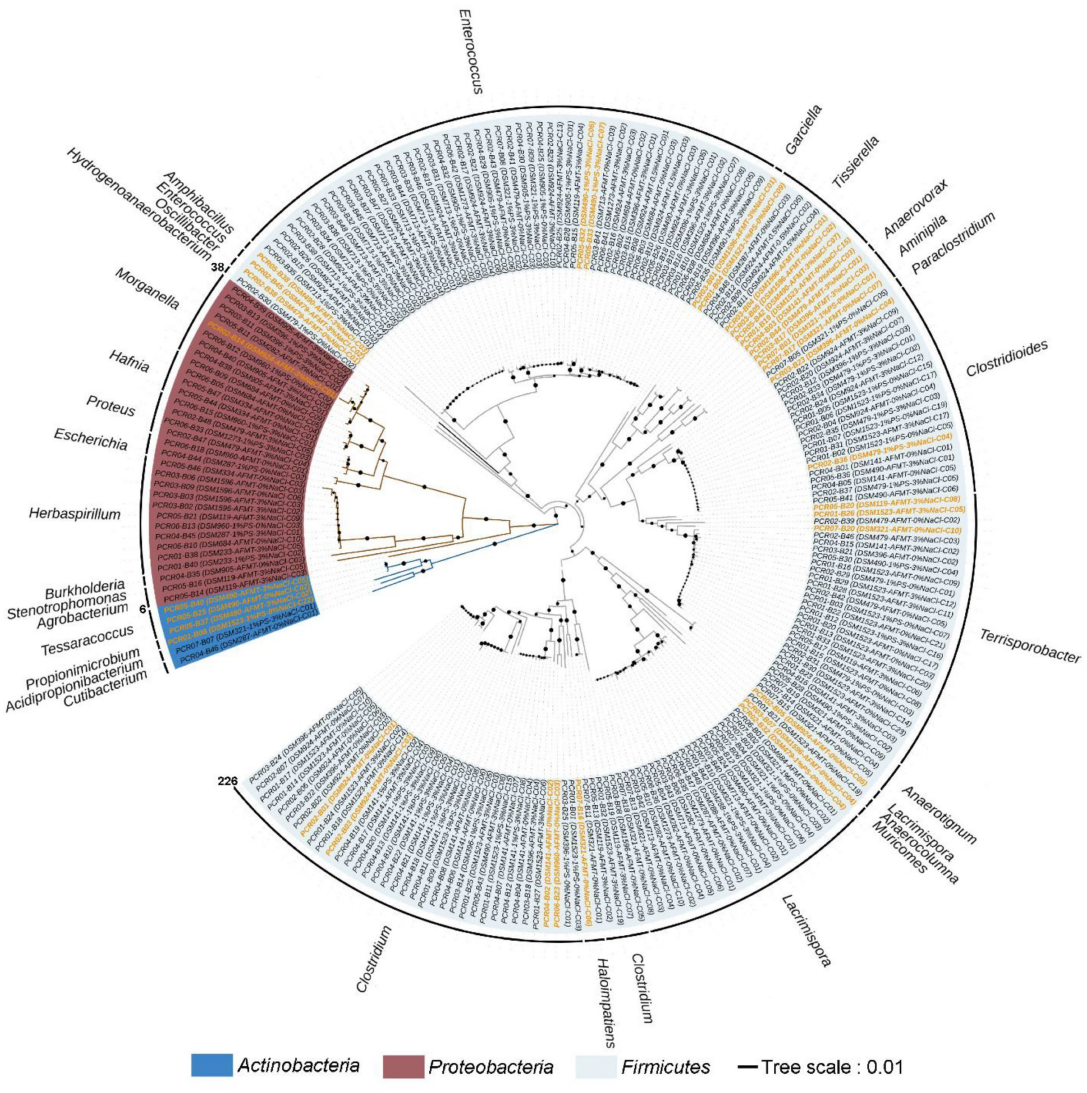
Neighbor-joining phylogenetic tree of strictly anaerobic and culturable bacteria isolated from the feces of *T. molitor* larvae based on 16S rRNA gene sequences; the effective sequence length was ~1,500 bp. This tree was constructed using the MEGA11 software package. Bar, 0.01 evolutionary distances. Sequences labeled with an orange color represent novel species or genera.

Comparing the species of isolated cultures with those previously identified in the fecal flora composition, including a control group fed bran and a treatment group fed polystyrene, showed that, at the phylum level, microorganisms from Actinobacteria, Firmicutes, and Proteobacteria had all been previously identified in the fecal flora composition (Figure 4A). However, at the class level, we have successfully enriched and isolated strains belonging to Actinomycetia (6 strains), Betaproteobacteria (12 strains), and Tissierellia (10 strains) through culturomics, which were not detected when the fecal bacterial phase was detected by 16S rRNA amplification sequencing technology (Figure 4B). Their abundance is not low, accounting for 2.7, 5.3, and 4.4% of the total bacteria, respectively (Figure 3B). Further analysis of these isolated cultures at the genus level revealed that a total of 24 genera were cultured that were not previously identified in the fecal flora composition. Among them, the 6 bacteria belonging to Actinomycetia can be further subdivided into *Acidipropionibacterium* (1 strain, 0.4%), *Cutibacterium* (1 strain, 0.4%), *Propionimicrobium* (1 strain, 0.4%), and *Tessaracoccus* (3 strains, 1.3%) and the 12 bacteria belonging to Betaproteobacteria can be further subdivided into *Burkholderia* (1 strains, 0.4%) and *Herbaspirillum* (11 strains, 4.9%), while the 10 bacteria belonging to *Tissierellia* are all *Tissierella* (4.4%) (Figures 3C, 4C). These results indicate that there is a rich diversity of culturable anaerobic microorganisms in the *T. molitor* gut (Figure 5).

### 3.3 Comparison of microbial diversity isolated from different media

**Figure 6:**
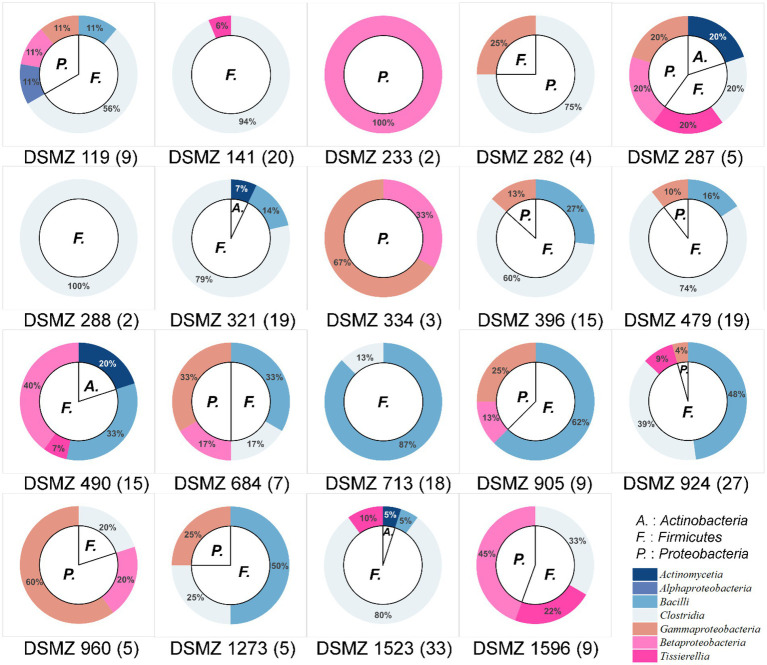
Community composition of cultivable bacteria on different culture media at the phylum level (inner) and class level (outer). The numbers in parentheses after the medium names indicate the number of strains isolated and purified in that medium.

**Figure 7:**
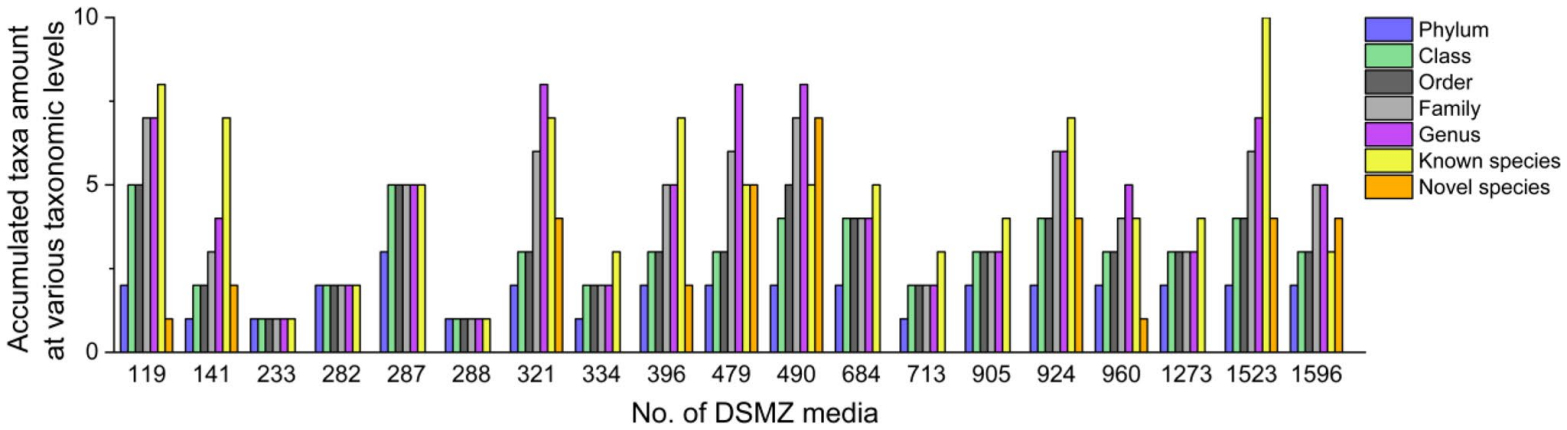
Diversity of bacteria cultured in different media at different taxonomic levels.

These 226 strains have been successfully enriched and isolated based on the culturomics approach in this study. The types and quantities of bacteria isolated from different media are also diverse (Figures 6, 7 and Supplementary Table S1). Notably, the roll-tube experiments for DSMZ 503 medium did not form any colonies.

There were artificial selection differences observed during the colony-picking process. In our study, only colonies displaying significant morphological differences and adhering to the tube wall after the rolling-tube experiment were manually picked, isolated, and cultured for subsequent analysis. Therefore, in some media, such as DSMZ 233, DSMZ 282, DSMZ 288, and DSMZ 334, only a few colonies with noticeable morphological differences were selected as representative strains and cultured in the medium (2, 4, 2, and 3 strains, respectively) based on subjective judgment. However, the diversity of strains cultured in each medium varied significantly, even at the phylum and class levels (Figure 6).

At the species level, the similarity between the strains isolated from each medium and the known species was compared, and those that were less than 98.65% were considered suspected novel species, while those that were ≧98.65% were identified as the same species. According to this judgment method, 226 purified strains isolated and cultured in this experiment belong to 42 known species, and 34 potentially represent novel species. The diversity of culturable strains obtained by using DSMZ 1523 medium was the highest, with 14 different species. It was followed by DSMZ 490, with 12 different species. The isolation results from DSMZ 321, DSMZ 924, DSMZ 479, DSMZ 119, DSMZ 141, and DSMZ 396 also showed high diversity, with 11, 11, 10, 9, 9, and 9 different species, respectively. In addition to these, only a few strains of different species were isolated and cultured in the rest of the medium through manual bacterial picking (Figure 7).

## 4 Discussion

### 4.1 *Escherichia–Shigella* phylogroup from Styrofoam-fed *Tenebrio molitor* larvae’s feces may harbor highly efficient ability for PS degradation

In this study, we changed the diet of *T. molitor* larvae to investigate the changes in their gut bacterial community. Compared with normal feeding (fed bran, control group, CG), the composition of the gut bacterial community of *T. molitor* larvae fed only with Styrofoam (treat group, TG) was significantly different from that of the CG (Figure 2). The results revealed a significant shift in the dominant phylogroup from *Lactococcus* (37.8%) to *Escherichia–Shigella* (54.7%) when comparing the feces of larvae fed with bran and Styrofoam, as analyzed through the bacterial 16S rRNA gene amplicon sequencing.

Analysis of changes in the differential microbiota, or dominant microbiota, in general can be considered to exhibit a high degree of adaptability and tolerance to environmental changes, or they may possess the ability to cope with and combat such changes (Zhong et al., 2022; Li et al., 2023). Based on the results of this study, the *Escherichia–Shigella* phylogroup emerges as the predominant group in the feces of *T. molitor* larvae exclusively fed with Styrofoam. It is speculated that the *Escherichia–Shigella* phylogroup identified in this study may possess the potential to biodegrade PS. Therefore, in future research, whether these strains are isolated from the surface of PS plastic waste exhibiting degradation traces in the natural environment or from within the digestive tracts of insects fed only with Styrofoam, their ability to degrade PS should be further examined, as they may hold significant potential.

### 4.2 The expanding variety and number of culturable anaerobic bacteria were obtained using the small-scale culturomics method

For environmental microbes, the majority are uncultured (Hugenholtz and Tyson, 2008). This may explain why highly efficient biodegrading strains have not yet been reported, either from the natural environment or in the guts of plastic-eating insects. In this study, we combined the strict anaerobic technique and the small-scale culturomics strategy to expand the variety and number of culturable anaerobic bacteria in the feces of Styrofoam-fed *T. molitor* larvae. We successfully isolated and cultured 226 strains of anaerobic bacteria, which belong to 29 genera and include 34 potential novel species. In addition, 24 genera identified through the culturomics method were not found in the results obtained from 16S rRNA gene amplicon sequencing (Figure 4C). These results show that, based on culturomics, we can maximize the variety and number of culturable strains.

The discovery of microorganisms in environmental samples, especially those with very low abundance, and trying to isolate and culture them not only helps us to deeply understand the community composition of microecosystems but also constitutes an essential step toward successfully uncovering the microecological balance of the gut. Expanding the variety and number of culturable strains based on culturomics will also greatly increase the possibility of finding potential strains, which is also a new idea for us to solve the plastic waste problem based on biotechnology.

### 4.3 Enriching and culturing specific species of microorganisms using a specific medium

Depending on the dependence of microorganism growth on the environment or the selectivity of the medium for specific types of microorganisms, different types of media are expected to culture different microorganisms (Dorofeev et al., 2014; Gonzalez and Aranda, 2023). This represents the core idea of the culturomics approach. Furthermore, there has been no research focusing on the isolation of gut anaerobes from Styrofoam-eating *T. molitor* larvae using strictly anaerobic techniques. Therefore, it is worth conducting our study to investigate the diversity of culturable anaerobic microbes in the gut of *T. molitor* larvae.

In this study, a total of 20 anaerobic media under four different conditions were used to study microbial diversity. A total of 226 anaerobic strains were enriched and isolated through serial dilution, the rolling-tube technique, and picking a single colony. Through 16S rRNA gene clone sequencing and phylogenetic analysis, these strains were identified as belonging to 42 known species and 34 potential novel species. These results highlight a significant diversity of culturable strains in the gut of *T. molitor*. It was also noteworthy that there were significant differences in the bacterial species cultured among the 19 types of media we employed (Figure 6).

Tracing back to the origin, we still want to use biotechnology to solve the problem of plastic waste accumulation. The microorganisms from Bacilli, Gammaproteobacteria, Alphaproteobacteria, and Betaproteobacteria have been reported in succession to have biodegradability of PS (Sekhar et al., 2016; Grgic et al., 2021; Jiang et al., 2021; Kim et al., 2021). In our study and the above inference, at the class level, microorganisms belonging to Gammaproteobacteria might have the potential to biodegrade PS because their abundance increases significantly after the host feeds on Styrofoam (Figure 2B). By further analyzing the variety and number of strains cultured in different media, we emphasized that, considering that the larger the number of microorganisms cultured, the more likely it is to be screened for true biodegradable strains, media DSMZ 119, DSMZ 141, DSMZ 321, DSMZ 396, DSMZ 479, DSMZ 490, DSMZ 924, and DSMZ 1523 are good choices for the enrichment of potential biodegradable bacteria in future studies. Because in our study, these media could culture the largest number of bacteria (Figure 7). If thought of in terms of bacterial species, then media DSMZ 684, DSMZ 905, and DSMZ 1273, especially DSMZ 334 and DSMZ 960, are good choices. Because these medium, in our study, successfully cultured Gammaproteobacteria strains, the medium DSMZ 334 and DSMZ 960 appear to be a specific medium for this type of bacteria, as bacteria from the genus Gammaproteobacteria make up 87 and 60% of the total, respectively.

### 4.4 The collection of culturable anaerobic bacteria from *Tenebrio molitor* larvae’s feces may provide a microbial resource for investigating the highly efficient PS biodegradation

The biodegradation of plastics by bacterial combinations, or mixed bacteria, has long been neglected. According to the study by Xiang et al. (2023), three strains with the biodegradability of PS were isolated from sediments seriously polluted by microplastics, and they were combined in pairs or mixed with three strains to evaluate their biodegradability of PS. The results showed that the degradation efficiency of bacterial combinations was significantly higher than that of single bacteria, indicating that effective combinations could greatly enhance the biodegradation efficiency. In our study, out of the 226 strains that were isolated, we might randomly combine some potential plastic-degrading strains, such as those belonging to *Escherichia–Shigella* and *Veillonella* (since they were dominant bacteria after feeding *T. molitor* with Styrofoam only for 6 months), or intentionally combine the isolated strains of specific species based on the abundance ratio from the fecal flora community structure (Figure 2C), in order to investigate the biodegradation efficiency of PS by bacterial combinations. This could also be a worthwhile endeavor for future research.

## 5 Conclusion

The gut microbial community composition of *T. molitor* larvae was significantly altered by feeding Styrofoam. In our study, the *Escherichia–Shigella* phylogroup emerges as the predominant group in the feces of *T. molitor* larvae exclusively fed with Styrofoam. Using a culturomics approach, we expanded the variety and number of culturable anaerobic bacteria in the feces of Styrofoam-fed *T. molitor* larvae. We successfully cultured strains that belonged to 24 genera that could not be detected by the bacterial 16S rRNA amplicon sequencing technique. Additionally, we present some personal views on the selection of medium for the enrichment of potential biodegradable bacteria in future studies. We propose that future studies aim to enrich and culture anaerobic bacteria with potential biodegradable PS plastics in the guts of *T. molitor* larvae. The media, such as DSMZ 119, DSMZ 141, DSMZ 321, DSMZ 396, DSMZ 479, DSMZ 490, DSMZ 924, and DSMZ 1523, are good choices because these media could culture the highest microbial diversity in our study. Other media, including DSMZ 334, DSMZ 684, DSMZ 905, DSMZ 960, and DSMZ 1273, are also good choices because, in our study, these media were able to culture some strains belonging to the same class that has been identified as having the ability to biodegrade PS. Additionally, it is also a promising direction to explore the mixed biodegradability of PS by combining some potential strains either randomly or based on the abundance ratio of microbial community composition.

## Data availability statement

The datasets presented in this study can be found in online repositories. The names of the repository/repositories and accession number(s) can be found in the article/Supplementary material.

## Ethics statement

The manuscript presents research on animals that do not require ethical approval for their study.

## Author contributions

JZ: Data curation, Formal analysis, Investigation, Methodology, Validation, Writing – original draft. XC: Funding acquisition, Investigation, Project administration, Supervision, Validation, Writing – review & editing. S-CC: Conceptualization, Funding acquisition, Investigation, Methodology, Project administration, Resources, Supervision, Validation, Writing – review & editing. WQ: Formal analysis, Investigation, Writing – original draft. JY: Funding acquisition, Project administration, Validation, Writing – review & editing. TG: Funding acquisition, Project administration, Supervision, Writing – review & editing. XW: Funding acquisition, Project administration, Supervision, Writing – review & editing.

## Footnotes

^1^ https://www.ezbiocloud.net/Identify

^2^ https://www.ncbi.nlm.nih.gov/
