# Supplementary material for: Diversity and community composition of strictly anaerobic and culturable bacteria from the feces of Styrofoam-fed Tenebrio molitor larvae: a culturomics-based study
Source: Front Microbiol. 2023 Dec 5;14:1309806. doi: 10.3389/fmicb.2023.1309806 (PMC10728288; doi:10.3389/fmicb.2023.1309806)
Supplement: Supplementary file 1 [file Data_Sheet_1.docx]

Supplementary Material

**Table S1 |** List of 39 media used by DSMZ to culture methane bacteria, the media selected media and the number of isolated strains cultured for this study

|  | Medium DSM number | The species and number of methane bacteria cultured in DSMZ by this medium | Select or not (√ or ×) | Number of isolated strains |
| --- | --- | --- | --- | --- |
| 1 | 119 | 38 strains *Methanobrevibacter smithii*, *Methanobrevibacter ruminantium*, *Methanobacterium palustre*, *Methanothermobacter wolfei*, *Methanococcus vannielii,* etc. | **√** | **9** |
| 2 | 120 | 12 strains *Methanosarcina thermophila*, *Methanosarcina mazei*, *Methanimicrococcus blatticola, etc.* | **×** |  |
| 3 | 141 | 39 strains *Methanobacterium arcticum*, *Methanobacterium ferruginis*, *Methanogenium cariaci*, *Methanolobus vulcani*, *Methanococcoides burtonii,* etc. | **√** | **20** |
| 4 | 161 | *Methanomicrobium mobile* | **×** |  |
| 5 | 203 | *Methanothermus fervidus*, *Methanothermus sociabilis* |  |  |
| 6 | 233 | *Methanolobus tindarius* | **√** | **2** |
| 7 | 274 | *Methanolacinia paynteri* | **×** |  |
| 8 | 282 | 11strains *Methanothermococcus okinawensis*, *Methanocaldococcus jannaschii*, *Methanocaldococcus vulcanius,* etc. | **√** | **4** |
| 9 | 287 | *Methanoculleus bourgensis*, *Methanoculleus chikugoensis* | **√** | **5** |
| 10 | 288 | *Methanococcus maripaludis* | **√** | **2** |
| 11 | 311 | *Methanobrevibacter filiformis* | **×** |  |
| 12 | 321 | *Methanocorpusculum aggregans* | **√** | **19** |
| 13 | 322 | *Methanosphaera stadtmanae* | **×** |  |
| 14 | 329 | *Methanohalophilus halophilus* | **×** |  |
| 15 | 334 | *Methanothrix soehngenii*, *Methanothrix thermoacetophila*, *Methanosaeta harundinacea* | **√** | **3** |
| 16 | 342 | *Methanobacterium alcaliphilum* | **×** |  |
| 17 | 348 | *Methanohalophilus portucalensis* | **×** |  |
| 18 | 375 | *Methanohalobium evestigatum* | **×** |  |
| 19 | 396 | *Methanosalsum zhilinae* | **√** | **15** |
| 20 | 404 | *Methanosphaera cuniculi* | **×** |  |
| 21 | 479 | *Methanohalophilus mahii* | **√** | **19** |
| 22 | 490 | *Methanolobus oregonensis* | **√** | **15** |
| 23 | 503 | *Methanomethylovorans hollandica*, *Methanomethylovorans uponensis* | **√** | **0** |
| 24 | 506 | *Methanobacterium espanolae*, *Methanobacterium veterum* | **×** |  |
| 25 | 511 | *Methanopyrus kandleri* | **×** |  |
| 26 | 684 | *Methanomethylovorans thermophila* | **√** | **7** |
| 27 | 713 | *Methanohalophilus euhalobius* | **√** | **18** |
| 28 | 734 | *Methanobrevibacter curvatus*, *Methanobrevibacter cuticularis* | **×** |  |
| 29 | 892 | *Methanocalculus pumilus* | **×** |  |
| 30 | 905 | *Methanocalculus halotolerans* | **√** | **9** |
| 31 | 924 | 6 strains *Methanofollis formosanus*, *Methanoculleus sediminis*, *Methanoculleus taiwanensis*, *Methanocalculus taiwanensis*, etc. | **√** | **27** |
| 32 | 960 | *Methanocella paludicola, Methanolinea tarda Methanolinea mesophila* | **√** | **5** |
| 33 | 1094 | *Methanobacterium paludis*, *Methanosphaerula palustris* | **×** |  |
| 34 | 1273 | *Methanospirillum lacunae* | **√** | **5** |
| 35 | 1318 | *Methanocella conradii* | **×** |  |
| 36 | 1329 | *Methanosaeta pelagica* | **×** |  |
| 37 | 1523 | *Methanofollis tationis*, *Methanofollis liminatans*, *Methanoculleus palmolei*, *Methanocorpusculum bavaricum*, *Methanocorpusculum sinense* | **√** | **33** |
| 38 | 1596 | *Methanocalculus alkaliphilus* | **√** | **9** |
| 39 | 1597 | *Methanosalsum natronophilum*, *Methanonatronarchaeum thermophilum* | **×** |  |

**Table S2 |** List of strains, their accession numbers and EZBioCloud identification results

| **No.** | **Clone name** | **Strain name** | **GenBank Accession Number** | **Top-hit taxon** | **Top-hit strain** | **Similarity (%)** | **Top-hit taxonomy-domain** | **Top-hit taxonomy-phylum** | **Top-hit taxonomy-class** | **Top-hit taxonomy-order** | **Top-hit taxonomy-family** | **Top-hit taxonomy-genus** | **Top-hit taxonomy-species** |
| --- | --- | --- | --- | --- | --- | --- | --- | --- | --- | --- | --- | --- | --- |
| 1 | PCR05-B12 | DSM119-AFMT-3%NaCl-C01 | OQ607915 | *Lacrimispora sphenoides* | JCM 1415 | 99.52 | Bacteria | Firmicutes | Clostridia | Clostridiales | Lachnospiraceae | *Lacrimispora* |  |
| 2 | PCR05-B13 | DSM119-AFMT-3%NaCl-C02 | OQ607916 | *Clostridium sartagoforme* | DSM 1292 | 99.86 | Bacteria | Firmicutes | Clostridia | Clostridiales | Clostridiaceae | *Clostridium* |  |
| 3 | PCR05-B14 | DSM119-AFMT-3%NaCl-C03 | OQ607917 | *Agrobacterium pusense* | LMG 25623 | 99.72 | Bacteria | Proteobacteria | Alphaproteobacteria | Rhizobiales | Rhizobiaceae | *Agrobacterium* |  |
| 4 | PCR05-B15 | DSM119-AFMT-3%NaCl-C04 | OQ607918 | *Enterococcus thailandicus* | DSM 21767 | 98.79 | Bacteria | Firmicutes | Bacilli | Lactobacillales | Enterococcaceae | *Enterococcus* |  |
| 5 | PCR05-B16 | DSM119-AFMT-3%NaCl-C05 | OQ607919 | *Stenotrophomonas pavanii* | DSM 25135 | 99.39 | Bacteria | Proteobacteria | Gammaproteobacteria | Lysobacterales | Lysobacteraceae | *Stenotrophomonas* |  |
| 6 | PCR05-B17 | DSM119-AFMT-3%NaCl-C06 | OQ607920 | *Terrisporobacter glycolicus* | DSM 1288 | 99.23 | Bacteria | Firmicutes | Clostridia | Clostridiales | Peptostreptococcaceae | *Terrisporobacter* |  |
| 7 | PCR05-B19 | DSM119-AFMT-3%NaCl-C07 | OQ607921 | *Clostridium sartagoforme* | DSM 1292 | 99.79 | Bacteria | Firmicutes | Clostridia | Clostridiales | Clostridiaceae | *Clostridium* |  |
| 8 | PCR05-B20 | DSM119-AFMT-3%NaCl-C08 | OQ607922 | *Terrisporobacter glycolicus* | DSM 1288 | 97.12 | Bacteria | Firmicutes | Clostridia | Clostridiales | Peptostreptococcaceae | *Terrisporobacter* |  |
| 9 | PCR05-B21 | DSM119-AFMT-3%NaCl-C09 | OQ607923 | *Herbaspirillum huttiense subsp. putei* | IAM 15032 | 99.86 | Bacteria | Proteobacteria | Betaproteobacteria | Burkholderiales | Oxalobacteraceae | *Herbaspirillum* | *Herbaspirillum huttiense* |
| 10 | PCR06-B33 | DSM1273-1%PS-3%NaCl-C03 | OQ608081 | *Proteus mirabilis* | ATCC 29906 | 99.59 | Bacteria | Proteobacteria | Gammaproteobacteria | Enterobacterales | Morganellaceae | *Proteus* |  |
| 11 | PCR06-B35 | DSM1273-AFMT-0%NaCl-C01 | OQ608082 | *Lacrimispora celerecrescens* | DSM 5628 | 99.59 | Bacteria | Firmicutes | Clostridia | Clostridiales | Lachnospiraceae | *Lacrimispora* |  |
| 12 | PCR06-B36 | DSM1273-AFMT-0%NaCl-C02 | OQ608083 | *Lacrimispora celerecrescens* | DSM 5628 | 99.59 | Bacteria | Firmicutes | Clostridia | Clostridiales | Lachnospiraceae | *Lacrimispora* |  |
| 13 | PCR06-B41 | DSM1273-AFMT-3%NaCl-C02 | OQ608084 | *Enterococcus thailandicus* | DSM 21767 | 99.73 | Bacteria | Firmicutes | Bacilli | Lactobacillales | Enterococcaceae | *Enterococcus* |  |
| 14 | PCR06-B42 | DSM1273-AFMT-3%NaCl-C03 | OQ608085 | *Enterococcus phoeniculicola* | ATCC BAA-412 | 99.93 | Bacteria | Firmicutes | Bacilli | Lactobacillales | Enterococcaceae | *Enterococcus* |  |
| 15 | PCR04-B18 | DSM141-1%PS-3%NaCl-C01 | OQ607777 | *Clostridium subterminale* | DSM 6970 | 99.72 | Bacteria | Firmicutes | Clostridia | Clostridiales | Clostridiaceae | *Clostridium* |  |
| 16 | PCR04-B19 | DSM141-1%PS-3%NaCl-C02 | OQ607778 | *Clostridium subterminale* | DSM 6970 | 99.72 | Bacteria | Firmicutes | Clostridia | Clostridiales | Clostridiaceae | *Clostridium* |  |
| 17 | PCR04-B20 | DSM141-1%PS-3%NaCl-C03 | OQ607779 | *Clostridium sulfidigenes* | SGB2 | 99.58 | Bacteria | Firmicutes | Clostridia | Clostridiales | Clostridiaceae | *Clostridium* |  |
| 18 | PCR04-B21 | DSM141-1%PS-3%NaCl-C04 | OQ607780 | *Clostridium subterminale* | DSM 6970 | 99.72 | Bacteria | Firmicutes | Clostridia | Clostridiales | Clostridiaceae | *Clostridium* |  |
| 19 | PCR04-B22 | DSM141-1%PS-3%NaCl-C05 | OQ607781 | *Clostridium subterminale* | DSM 6970 | 99.72 | Bacteria | Firmicutes | Clostridia | Clostridiales | Clostridiaceae | *Clostridium* |  |
| 20 | PCR04-B10 | DSM141-1%PS-3%NaCl-C06 | OQ607770 | *Clostridium subterminale* | DSM 6970 | 99.65 | Bacteria | Firmicutes | Clostridia | Clostridiales | Clostridiaceae | *Clostridium* |  |
| 21 | PCR04-B11 | DSM141-1%PS-3%NaCl-C07 | OQ607771 | *Clostridium subterminale* | DSM 6970 | 99.72 | Bacteria | Firmicutes | Clostridia | Clostridiales | Clostridiaceae | *Clostridium* |  |
| 22 | PCR04-B12 | DSM141-1%PS-3%NaCl-C08 | OQ607772 | *Clostridium sporogenes* | DSM 795 | 99.72 | Bacteria | Firmicutes | Clostridia | Clostridiales | Clostridiaceae | *Clostridium* |  |
| 23 | PCR04-B13 | DSM141-1%PS-3%NaCl-C09 | OQ607773 | *Clostridium subterminale* | DSM 6970 | 99.65 | Bacteria | Firmicutes | Clostridia | Clostridiales | Clostridiaceae | *Clostridium* |  |
| 24 | PCR04-B01 | DSM141-AFMT-0%NaCl-C01 | OQ607762 | *Clostridioides mangenotii* | DSM 1289 | 99.37 | Bacteria | Firmicutes | Clostridia | Clostridiales | Peptostreptococcaceae | *Clostridioides* |  |
| 25 | PCR04-B02 | DSM141-AFMT-0%NaCl-C02 | OQ607763 | *Clostridium botulinum* | ATCC 25763 | 97.00 | Bacteria | Firmicutes | Clostridia | Clostridiales | Clostridiaceae | *Clostridium* |  |
| 26 | PCR04-B03 | DSM141-AFMT-0%NaCl-C03 | OQ607764 | *Urmitella timonensis* | Marseille-P2918 | 96.97 | Bacteria | Firmicutes | Tissierellia | Tissierellales | Tissierellaceae | *Tissierella* |  |
| 27 | PCR04-B04 | DSM141-AFMT-0%NaCl-C04 | OQ607765 | *Clostridium sporogenes* | DSM 795 | 99.86 | Bacteria | Firmicutes | Clostridia | Clostridiales | Clostridiaceae | *Clostridium* |  |
| 28 | PCR04-B05 | DSM141-AFMT-0%NaCl-C05 | OQ607766 | *Clostridioides mangenotii* | DSM 1289 | 99.30 | Bacteria | Firmicutes | Clostridia | Clostridiales | Peptostreptococcaceae | *Clostridioides* |  |
| 29 | PCR04-B06 | DSM141-AFMT-0%NaCl-C06 | OQ607767 | *Clostridium sporogenes* | DSM 795 | 99.79 | Bacteria | Firmicutes | Clostridia | Clostridiales | Clostridiaceae | *Clostridium* |  |
| 30 | PCR04-B07 | DSM141-AFMT-0%NaCl-C07 | OQ607768 | *Clostridium sporogenes* | DSM 795 | 99.58 | Bacteria | Firmicutes | Clostridia | Clostridiales | Clostridiaceae | *Clostridium* |  |
| 31 | PCR04-B08 | DSM141-AFMT-0%NaCl-C08 | OQ607769 | *Clostridium pascui* | DSM 10365 | 98.95 | Bacteria | Firmicutes | Clostridia | Clostridiales | Clostridiaceae | *Clostridium* |  |
| 32 | PCR04-B15 | DSM141-AFMT-3%NaCl-C02 | OQ607774 | *Terrisporobacter othiniensis* | 08-306576 | 99.13 | Bacteria | Firmicutes | Clostridia | Clostridiales | Peptostreptococcaceae | *Terrisporobacter* |  |
| 33 | PCR04-B16 | DSM141-AFMT-3%NaCl-C03 | OQ607775 | *Terrisporobacter glycolicus* | DSM 1288 | 99.30 | Bacteria | Firmicutes | Clostridia | Clostridiales | Peptostreptococcaceae | *Terrisporobacter* |  |
| 34 | PCR04-B17 | DSM141-AFMT-3%NaCl-C04 | OQ607776 | *Clostridium sulfidigenes* | SGB2 | 99.72 | Bacteria | Firmicutes | Clostridia | Clostridiales | Clostridiaceae | *Clostridium* |  |
| 35 | PCR01-B01 | DSM1523-1%PS-0%NaCl-C03 | OQ147097 | *Clostridium cochlearium* | NCTC 13027 | 99.93 | Bacteria | Firmicutes | Clostridia | Clostridiales | Clostridiaceae | *Clostridium* |  |
| 36 | PCR01-B02 | DSM1523-1%PS-0%NaCl-C05 | OQ147098 | *Clostridioides mangenotii* | DSM 1289 | 99.37 | Bacteria | Firmicutes | Clostridia | Clostridiales | Peptostreptococcaceae | *Clostridioides* |  |
| 37 | PCR01-B03 | DSM1523-1%PS-0%NaCl-C07 | OQ147099 | *Terrisporobacter glycolicus* | DSM 1288 | 99.23 | Bacteria | Firmicutes | Clostridia | Clostridiales | Peptostreptococcaceae | *Terrisporobacter* |  |
| 38 | PCR01-B04 | DSM1523-1%PS-0%NaCl-C09 | OQ147100 | *Tissierella carlieri* | LBN 295 | 93.78 | Bacteria | Firmicutes | Tissierellia | Tissierellales | Tissierellaceae | *Tissierella* |  |
| 39 | PCR01-B05 | DSM1523-1%PS-0%NaCl-C15 | OQ147101 | *Clostridioides mangenotii* | DSM 1289 | 99.30 | Bacteria | Firmicutes | Clostridia | Clostridiales | Peptostreptococcaceae | *Clostridioides* |  |
| 40 | PCR01-B06 | DSM1523-1%PS-0%NaCl-C17 | OQ147102 | *Clostridioides mangenotii* | DSM 1289 | 99.44 | Bacteria | Firmicutes | Clostridia | Clostridiales | Peptostreptococcaceae | *Clostridioides* |  |
| 41 | PCR01-B07 | DSM1523-1%PS-0%NaCl-C19 | OQ147103 | *Clostridioides mangenotii* | DSM 1289 | 99.37 | Bacteria | Firmicutes | Clostridia | Clostridiales | Peptostreptococcaceae | *Clostridioides* |  |
| 42 | PCR01-B08 | DSM1523-1%PS-0%NaCl-C22 | OQ147104 | *Propionibacterium westphaliense* | 1a7I-CH12an | 94.73 | Bacteria | Actinobacteria | Actinomycetia | Propionibacteriales | Propionibacteriaceae | *Propionimicrobium* |  |
| 43 | PCR01-B09 | DSM1523-1%PS-3%NaCl-C02 | OQ147105 | *Clostridium senegalense* | JC122 | 99.79 | Bacteria | Firmicutes | Clostridia | Clostridiales | Clostridiaceae | *Clostridium* |  |
| 44 | PCR01-B10 | DSM1523-1%PS-3%NaCl-C07 | OQ147106 | *Enterococcus avium* | ATCC 14025 | 99.80 | Bacteria | Firmicutes | Bacilli | Lactobacillales | Enterococcaceae | *Enterococcus* |  |
| 45 | PCR01-B11 | DSM1523-1%PS-3%NaCl-C15 | OQ147107 | *Clostridium sporogenes* | DSM 795(T) | 99.72 | Bacteria | Firmicutes | Clostridia | Clostridiales | Clostridiaceae | *Clostridium* |  |
| 46 | PCR01-B12 | DSM1523-1%PS-3%NaCl-C16 | OQ147108 | *Terrisporobacter glycolicus* | DSM 1288 | 99.30 | Bacteria | Firmicutes | Clostridia | Clostridiales | Peptostreptococcaceae | *Terrisporobacter* |  |
| 47 | PCR01-B13 | DSM1523-AFMT-0%NaCl-C03 | OQ147109 | *Terrisporobacter glycolicus* | DSM 1288 | 99.23 | Bacteria | Firmicutes | Clostridia | Clostridiales | Peptostreptococcaceae | *Terrisporobacter* |  |
| 48 | PCR01-B14 | DSM1523-AFMT-0%NaCl-C05 | OQ147110 | *Clostridium argentinense* | ATCC 27322 | 99.09 | Bacteria | Firmicutes | Clostridia | Clostridiales | Clostridiaceae | *Clostridium* |  |
| 49 | PCR01-B15 | DSM1523-AFMT-0%NaCl-C08 | OQ147111 | *Terrisporobacter glycolicus* | DSM 1288 | 99.23 | Bacteria | Firmicutes | Clostridia | Clostridiales | Peptostreptococcaceae | *Terrisporobacter* |  |
| 50 | PCR01-B16 | DSM1523-AFMT-0%NaCl-C09 | OQ147112 | *Terrisporobacter glycolicus* | DSM 1288 | 99.23 | Bacteria | Firmicutes | Clostridia | Clostridiales | Peptostreptococcaceae | *Terrisporobacter* |  |
| 51 | PCR01-B17 | DSM1523-AFMT-0%NaCl-C10 | OQ147113 | *Clostridium argentinense* | ATCC 27322 | 99.09 | Bacteria | Firmicutes | Clostridia | Clostridiales | Clostridiaceae | *Clostridium* |  |
| 52 | PCR01-B18 | DSM1523-AFMT-0%NaCl-C14 | OQ147114 | *Clostridium argentinense* | ATCC 27322 | 99.65 | Bacteria | Firmicutes | Clostridia | Clostridiales | Clostridiaceae | *Clostridium* |  |
| 53 | PCR01-B19 | DSM1523-AFMT-0%NaCl-C15 | OQ147115 | *Urmitella timonensis* | Marseille-P2918 | 96.97 | Bacteria | Firmicutes | Tissierellia | Tissierellales | Tissierellaceae | *Tissierella* |  |
| 54 | PCR01-B20 | DSM1523-AFMT-0%NaCl-C17 | OQ147116 | *Terrisporobacter glycolicus* | DSM 1288 | 99.30 | Bacteria | Firmicutes | Clostridia | Clostridiales | Peptostreptococcaceae | *Terrisporobacter* |  |
| 55 | PCR01-B21 | DSM1523-AFMT-0%NaCl-C19 | OQ147117 | *Anaerocolumna aminovalerica* | DSM 1283 | 99.45 | Bacteria | Firmicutes | Clostridia | Clostridiales | Lachnospiraceae | *Anaerocolumna* |  |
| 56 | PCR01-B22 | DSM1523-AFMT-0%NaCl-C21 | OQ147118 | *Terrisporobacter glycolicus* | DSM 1288 | 99.23 | Bacteria | Firmicutes | Clostridia | Clostridiales | Peptostreptococcaceae | *Terrisporobacter* |  |
| 57 | PCR01-B23 | DSM1523-AFMT-0%NaCl-C23 | OQ147119 | *Terrisporobacter glycolicus* | DSM 1288 | 99.30 | Bacteria | Firmicutes | Clostridia | Clostridiales | Peptostreptococcaceae | *Terrisporobacter* |  |
| 58 | PCR01-B24 | DSM1523-AFMT-3%NaCl-C02 | OQ147120 | *Clostridium argentinense* | ATCC 27322 | 99.51 | Bacteria | Firmicutes | Clostridia | Clostridiales | Clostridiaceae | *Clostridium* |  |
| 59 | PCR01-B25 | DSM1523-AFMT-3%NaCl-C03 | OQ147121 | *Clostridium sporogenes* | DSM 795 | 99.79 | Bacteria | Firmicutes | Clostridia | Clostridiales | Clostridiaceae | *Clostridium* |  |
| 60 | PCR01-B26 | DSM1523-AFMT-3%NaCl-C05 | OQ147122 | *Terrisporobacter petrolearius* | LAM0A37 | 96.54 | Bacteria | Firmicutes | Clostridia | Clostridiales | Peptostreptococcaceae | *Terrisporobacter* |  |
| 61 | PCR01-B27 | DSM1523-AFMT-3%NaCl-C06 | OQ147123 | *Clostridium sporogenes* | DSM 795 | 99.79 | Bacteria | Firmicutes | Clostridia | Clostridiales | Clostridiaceae | *Clostridium* |  |
| 62 | PCR01-B28 | DSM1523-AFMT-3%NaCl-C11 | OQ147124 | *Terrisporobacter othiniensis* | 08-306576 | 99.20 | Bacteria | Firmicutes | Clostridia | Clostridiales | Peptostreptococcaceae | *Terrisporobacter* |  |
| 63 | PCR01-B29 | DSM1523-AFMT-3%NaCl-C12 | OQ147125 | *Terrisporobacter glycolicus* | DSM 1288 | 99.09 | Bacteria | Firmicutes | Clostridia | Clostridiales | Peptostreptococcaceae | *Terrisporobacter* |  |
| 64 | PCR01-B30 | DSM1523-AFMT-3%NaCl-C14 | OQ147126 | *Terrisporobacter glycolicus* | DSM 1288 | 99.23 | Bacteria | Firmicutes | Clostridia | Clostridiales | Peptostreptococcaceae | *Terrisporobacter* |  |
| 65 | PCR01-B31 | DSM1523-AFMT-3%NaCl-C17 | OQ147127 | *Clostridioides mangenotii* | DSM 1289 | 99.37 | Bacteria | Firmicutes | Clostridia | Clostridiales | Peptostreptococcaceae | *Clostridioides* |  |
| 66 | PCR01-B32 | DSM1523-AFMT-3%NaCl-C19 | OQ147128 | *Clostridium sartagoforme* | DSM 1292 | 99.86 | Bacteria | Firmicutes | Clostridia | Clostridiales | Clostridiaceae | *Clostridium* |  |
| 67 | PCR01-B33 | DSM1523-AFMT-3%NaCl-C20 | OQ147129 | *Terrisporobacter glycolicus* | DSM 1288 | 99.23 | Bacteria | Firmicutes | Clostridia | Clostridiales | Peptostreptococcaceae | *Terrisporobacter* |  |
| 68 | PCR03-B04 | DSM1596-AFMT-0%NaCl-C01 | OQ607132 | *Tissierella hominis* | NSJ-26 | 93.40 | Bacteria | Firmicutes | Tissierellia | Tissierellales | Tissierellaceae | *Tissierella* |  |
| 69 | PCR03-B05 | DSM1596-AFMT-0%NaCl-C02 | OQ607133 | *Tissierella creatinini* | DSM 9508 | 93.26 | Bacteria | Firmicutes | Tissierellia | Tissierellales | Tissierellaceae | *Tissierella* |  |
| 70 | PCR03-B06 | DSM1596-AFMT-0%NaCl-C03 | OQ607134 | *Herbaspirillum huttiense subsp. huttiense* | ATCC 14670 | 99.93 | Bacteria | Proteobacteria | Betaproteobacteria | Burkholderiales | Oxalobacteraceae | *Herbaspirillum* | *Herbaspirillum huttiense* |
| 71 | PCR03-B07 | DSM1596-AFMT-0%NaCl-C04 | OQ607135 | *Faecalicatena contorta* | DSM 3982 | 97.52 | Bacteria | Firmicutes | Clostridia | Clostridiales | Lachnospiraceae | *Muricomes* |  |
| 72 | PCR03-B08 | DSM1596-AFMT-0%NaCl-C05 | OQ607136 | *Clostridium intestinale* | DSM 6191 | 99.42 | Bacteria | Firmicutes | Clostridia | Clostridiales | Clostridiaceae | *Clostridium* |  |
| 73 | PCR03-B09 | DSM1596-AFMT-0%NaCl-C06 | OQ607137 | *Herbaspirillum huttiense subsp. huttiense* | ATCC 14670 | 99.86 | Bacteria | Proteobacteria | Betaproteobacteria | Burkholderiales | Oxalobacteraceae | *Herbaspirillum* | *Herbaspirillum huttiense* |
| 74 | PCR03-B01 | DSM1596-AFMT-3%NaCl-C01 | OQ607129 | *Garciella nitratireducens* | DSM 15102 | 91.45 | Bacteria | Firmicutes | Clostridia | Clostridiales | Eubacteriaceae | *Garciella* |  |
| 75 | PCR03-B02 | DSM1596-AFMT-3%NaCl-C02 | OQ607130 | *Herbaspirillum huttiense subsp. putei* | IAM 15032 | 99.86 | Bacteria | Proteobacteria | Betaproteobacteria | Burkholderiales | Oxalobacteraceae | *Herbaspirillum* | *Herbaspirillum huttiense* |
| 76 | PCR03-B03 | DSM1596-AFMT-3%NaCl-C03 | OQ607131 | *Herbaspirillum huttiense subsp. huttiense* | ATCC 14670 | 100.00 | Bacteria | Proteobacteria | Betaproteobacteria | Burkholderiales | Oxalobacteraceae | *Herbaspirillum* | *Herbaspirillum huttiense* |
| 77 | PCR01-B40 | DSM233-1%PS-3%NaCl-C02 | OQ147131 | *Herbaspirillum huttiense subsp. huttiense* | ATCC 14670 | 99.86 | Bacteria | Proteobacteria | Betaproteobacteria | Burkholderiales | Oxalobacteraceae | *Herbaspirillum* | *Herbaspirillum huttiense* |
| 78 | PCR01-B38 | DSM233-AFMT-3%NaCl-C05 | OQ147130 | *Herbaspirillum huttiense subsp. huttiense* | ATCC 14670 | 99.93 | Bacteria | Proteobacteria | Betaproteobacteria | Burkholderiales | Oxalobacteraceae | *Herbaspirillum* | *Herbaspirillum huttiense* |
| 79 | PCR05-B04 | DSM282-AFMT-0%NaCl-C04 | OQ607911 | *Lacrimispora celerecrescens* | DSM 5628 | 99.24 | Bacteria | Firmicutes | Clostridia | Clostridiales | Lachnospiraceae | *Lacrimispora* |  |
| 80 | PCR05-B06 | DSM282-AFMT-0%NaCl-C06 | OQ607912 | *Lacrimispora celerecrescens* | DSM 5628 | 99.45 | Bacteria | Firmicutes | Clostridia | Clostridiales | Lachnospiraceae | *Lacrimispora* |  |
| 81 | PCR05-B10 | DSM282-AFMT-0%NaCl-C10 | OQ607913 | *Lacrimispora celerecrescens* | DSM 5628 | 99.66 | Bacteria | Firmicutes | Clostridia | Clostridiales | Lachnospiraceae | *Lacrimispora* |  |
| 82 | PCR05-B11 | DSM282-AFMT-3%NaCl-C01 | OQ607914 | *Morganella morganii subsp. morganii* | ATCC 25830 | 99.32 | Bacteria | Proteobacteria | Gammaproteobacteria | Enterobacterales | Morganellaceae | *Morganella* | *Morganella morganii* |
| 83 | PCR04-B44 | DSM287-1%PS-0%NaCl-C02 | OQ607791 | *Shigella flexneri* | ATCC 29903 | 99.73 | Bacteria | Proteobacteria | Gammaproteobacteria | Enterobacterales | Enterobacteriaceae | *Escherichia* |  |
| 84 | PCR04-B45 | DSM287-1%PS-3%NaCl-C01 | OQ607792 | *Herbaspirillum huttiense subsp. putei* | IAM 15032 | 100.00 | Bacteria | Proteobacteria | Betaproteobacteria | Burkholderiales | Oxalobacteraceae | *Herbaspirillum* | *Herbaspirillum huttiense* |
| 85 | PCR04-B46 | DSM287-AFMT-0%NaCl-C01 | OQ607793 | *Cutibacterium acnes subsp. acnes* | DSM 1897 | 99.65 | Bacteria | Actinobacteria | Actinomycetia | Propionibacteriales | Propionibacteriaceae | *Cutibacterium* | *Cutibacterium acnes* |
| 86 | PCR04-B47 | DSM287-AFMT-0%NaCl-C02 | OQ607794 | *Lacrimispora celerecrescens* | DSM 5628 | 99.66 | Bacteria | Firmicutes | Clostridia | Clostridiales | Lachnospiraceae | *Lacrimispora* |  |
| 87 | PCR04-B48 | DSM287-AFMT-0%NaCl-C03 | OQ607795 | *Tissierella carlieri* | LBN 295 | 99.50 | Bacteria | Firmicutes | Tissierellia | Tissierellales | Tissierellaceae | *Tissierella* |  |
| 88 | PCR01-B42 | DSM288-1%PS-3%NaCl-C01 | OQ147132 | *Lacrimispora celerecrescens* | DSM 5628 | 99.72 | Bacteria | Firmicutes | Clostridia | Clostridiales | Lachnospiraceae | *Lacrimispora* |  |
| 89 | PCR01-B48 | DSM288-AFMT-0%NaCl-C07 | OQ147133 | *Lacrimispora celerecrescens* | DSM 5628 | 99.79 | Bacteria | Firmicutes | Clostridia | Clostridiales | Lachnospiraceae | *Lacrimispora* |  |
| 90 | PCR07-B01 | DSM321-1%PS-0%NaCl-C01 | OQ608735 | *Aminipila butyrica* | FH042 | 96.11 | Bacteria | Firmicutes | Clostridia | Clostridiales | Mogibacterium_f | *Aminipila* |  |
| 91 | PCR07-B02 | DSM321-1%PS-0%NaCl-C02 | OQ608736 | *Hungatella xylanolytica* | X5-1 | 99.24 | Bacteria | Firmicutes | Clostridia | Clostridiales | Lachnospiraceae | *Lacrimispora* |  |
| 92 | PCR07-B03 | DSM321-1%PS-0%NaCl-C03 | OQ608737 | *Hungatella xylanolytica* | X5-1 | 99.31 | Bacteria | Firmicutes | Clostridia | Clostridiales | Lachnospiraceae | *Lacrimispora* |  |
| 93 | PCR07-B04 | DSM321-1%PS-0%NaCl-C04 | OQ608738 | *Hungatella xylanolytica* | X5-1 | 99.04 | Bacteria | Firmicutes | Clostridia | Clostridiales | Lachnospiraceae | *Lacrimispora* |  |
| 94 | PCR07-B05 | DSM321-1%PS-0%NaCl-C05 | OQ608739 | *Paraclostridium benzoelyticum* | JC272 | 99.72 | Bacteria | Firmicutes | Clostridia | Clostridiales | Peptostreptococcaceae | *Paraclostridium* |  |
| 95 | PCR07-B06 | DSM321-1%PS-0%NaCl-C06 | OQ608740 | *Hungatella xylanolytica* | X5-1 | 99.31 | Bacteria | Firmicutes | Clostridia | Clostridiales | Lachnospiraceae | *Lacrimispora* |  |
| 96 | PCR07-B07 | DSM321-1%PS-3%NaCl-C01 | OQ608741 | *Acidipropionibacterium acidipropionici* | CGMCC 1.2230 | 99.38 | Bacteria | Actinobacteria | Actinomycetia | Propionibacteriales | Propionibacteriaceae | *Acidipropionibacterium* |  |
| 97 | PCR07-B08 | DSM321-1%PS-3%NaCl-C02 | OQ608742 | *Enterococcus gallinarum* | NBRC 100675 | 99.87 | Bacteria | Firmicutes | Bacilli | Lactobacillales | Enterococcaceae | *Enterococcus* |  |
| 98 | PCR07-B09 | DSM321-1%PS-3%NaCl-C03 | OQ608743 | *Enterococcus gallinarum* | NBRC 100675 | 99.93 | Bacteria | Firmicutes | Bacilli | Lactobacillales | Enterococcaceae | *Enterococcus* |  |
| 99 | PCR07-B10 | DSM321-1%PS-3%NaCl-C04 | OQ608744 | *Lacrimispora celerecrescens* | DSM 5628 | 99.72 | Bacteria | Firmicutes | Clostridia | Clostridiales | Lachnospiraceae | *Lacrimispora* |  |
| 100 | PCR07-B11 | DSM321-AFMT-0%NaCl-C01 | OQ608745 | *Haloimpatiens massiliensis* | Mt13 | 99.37 | Bacteria | Firmicutes | Clostridia | Clostridiales | Clostridiaceae | *Haloimpatiens* |  |
| 101 | PCR07-B13 | DSM321-AFMT-0%NaCl-C03 | OQ608746 | *Lacrimispora celerecrescens* | DSM 5628 | 99.72 | Bacteria | Firmicutes | Clostridia | Clostridiales | Lachnospiraceae | *Lacrimispora* |  |
| 102 | PCR07-B14 | DSM321-AFMT-0%NaCl-C04 | OQ608747 | *Anaerotignum propionicum* | DSM 1682 | 99.73 | Bacteria | Firmicutes | Clostridia | Clostridiales | Lachnospiraceae | *Anaerotignum* |  |
| 103 | PCR07-B15 | DSM321-AFMT-0%NaCl-C05 | OQ608748 | *Anaerotignum propionicum* | DSM 1682 | 99.66 | Bacteria | Firmicutes | Clostridia | Clostridiales | Lachnospiraceae | *Anaerotignum* |  |
| 104 | PCR07-B16 | DSM321-AFMT-0%NaCl-C06 | OQ608749 | *Haloimpatiens massiliensis* | Mt13 | 96.22 | Bacteria | Firmicutes | Clostridia | Clostridiales | Clostridiaceae | *Haloimpatiens* |  |
| 105 | PCR07-B17 | DSM321-AFMT-0%NaCl-C07 | OQ608750 | *Aminipila butyrica* | FH042 | 96.11 | Bacteria | Firmicutes | Clostridia | Clostridiales | Mogibacterium_f | *Aminipila* |  |
| 106 | PCR07-B18 | DSM321-AFMT-0%NaCl-C08 | OQ608751 | *Lacrimispora celerecrescens* | DSM 5628 | 99.72 | Bacteria | Firmicutes | Clostridia | Clostridiales | Lachnospiraceae | *Lacrimispora* |  |
| 107 | PCR07-B19 | DSM321-AFMT-0%NaCl-C09 | OQ608752 | *Anaerotignum propionicum* | DSM 1682 | 99.66 | Bacteria | Firmicutes | Clostridia | Clostridiales | Lachnospiraceae | *Anaerotignum* |  |
| 108 | PCR07-B20 | DSM321-AFMT-0%NaCl-C10 | OQ608753 | *Terrisporobacter othiniensis* | 08-306576 | 96.87 | Bacteria | Firmicutes | Clostridia | Clostridiales | Peptostreptococcaceae | *Terrisporobacter* |  |
| 109 | PCR05-B44 | DSM334-AFMT-0%NaCl-C01 | OQ607939 | *Hafnia paralvei* | ATCC 29927 | 99.66 | Bacteria | Proteobacteria | Gammaproteobacteria | Enterobacterales | Hafniaceae | *Hafnia* |  |
| 110 | PCR05-B46 | DSM334-AFMT-0%NaCl-C03 | OQ607940 | *Herbaspirillum huttiense subsp. huttiense* | ATCC 14670 | 99.86 | Bacteria | Proteobacteria | Betaproteobacteria | Burkholderiales | Oxalobacteraceae | *Herbaspirillum* | *Herbaspirillum huttiense* |
| 111 | PCR05-B47 | DSM334-AFMT-0%NaCl-C04 | OQ607941 | *Hafnia paralvei* | ATCC 29927 | 99.45 | Bacteria | Proteobacteria | Gammaproteobacteria | Enterobacterales | Hafniaceae | *Hafnia* |  |
| 112 | PCR03-B25 | DSM396-1%PS-0%NaCl-C01 | OQ607152 | *Clostridium malenominatum* | DSM 1127 | 100.00 | Bacteria | Firmicutes | Clostridia | Clostridiales | Clostridiaceae | *Clostridium* |  |
| 113 | PCR03-B10 | DSM396-1%PS-3%NaCl-C01 | OQ607138 | *Enterococcus avium* | ATCC 14025 | 99.80 | Bacteria | Firmicutes | Bacilli | Lactobacillales | Enterococcaceae | *Enterococcus* |  |
| 114 | PCR03-B11 | DSM396-1%PS-3%NaCl-C02 | OQ607139 | *Morganella morganii subsp. sibonii* | DSM 14850 | 99.63 | Bacteria | Proteobacteria | Gammaproteobacteria | Enterobacterales | Morganellaceae | *Morganella* | *Morganella morganii* |
| 115 | PCR03-B12 | DSM396-1%PS-3%NaCl-C03 | OQ607140 | *Clostridioides mangenotii* | DSM 1289 | 99.02 | Bacteria | Firmicutes | Clostridia | Clostridiales | Peptostreptococcaceae | *Clostridioides* |  |
| 116 | PCR03-B13 | DSM396-1%PS-3%NaCl-C04 | OQ607141 | *Morganella morganii subsp. sibonii* | DSM 14850 | 99.78 | Bacteria | Proteobacteria | Gammaproteobacteria | Enterobacterales | Morganellaceae | *Morganella* | *Morganella morganii* |
| 117 | PCR03-B14 | DSM396-1%PS-3%NaCl-C05 | OQ607142 | *Clostridium sporogenes* | DSM 795 | 99.72 | Bacteria | Firmicutes | Clostridia | Clostridiales | Clostridiaceae | *Clostridium* |  |
| 118 | PCR03-B21 | DSM396-AFMT-0%NaCl-C02 | OQ607148 | *Terrisporobacter glycolicus* | DSM 1288 | 98.95 | Bacteria | Firmicutes | Clostridia | Clostridiales | Peptostreptococcaceae | *Terrisporobacter* |  |
| 119 | PCR03-B22 | DSM396-AFMT-0%NaCl-C03 | OQ607149 | *Clostridium argentinense* | ATCC 27322 | 98.88 | Bacteria | Firmicutes | Clostridia | Clostridiales | Clostridiaceae | *Clostridium* |  |
| 120 | PCR03-B23 | DSM396-AFMT-0%NaCl-C04 | OQ607150 | *Clostridioides mangenotii* | DSM 1289 | 97.47 | Bacteria | Firmicutes | Clostridia | Clostridiales | Peptostreptococcaceae | *Clostridioides* |  |
| 121 | PCR03-B24 | DSM396-AFMT-0%NaCl-C05 | OQ607151 | *Clostridium argentinense* | ATCC 27322 | 99.02 | Bacteria | Firmicutes | Clostridia | Clostridiales | Clostridiaceae | *Clostridium* |  |
| 122 | PCR03-B15 | DSM396-AFMT-3%NaCl-C01 | OQ607143 | *Enterococcus avium* | ATCC 14025 | 99.73 | Bacteria | Firmicutes | Bacilli | Lactobacillales | Enterococcaceae | *Enterococcus* |  |
| 123 | PCR03-B16 | DSM396-AFMT-3%NaCl-C02 | OQ607144 | *Enterococcus avium* | ATCC 14025 | 99.53 | Bacteria | Firmicutes | Bacilli | Lactobacillales | Enterococcaceae | *Enterococcus* |  |
| 124 | PCR03-B17 | DSM396-AFMT-3%NaCl-C03 | OQ607145 | *Aminipila butyrica* | FH042 | 96.25 | Bacteria | Firmicutes | Clostridia | Clostridiales | Mogibacterium_f | *Aminipila* |  |
| 125 | PCR03-B18 | DSM396-AFMT-3%NaCl-C04 | OQ607146 | *Clostridium sporogenes* | DSM 795 | 100.00 | Bacteria | Firmicutes | Clostridia | Clostridiales | Clostridiaceae | *Clostridium* |  |
| 126 | PCR03-B19 | DSM396-AFMT-3%NaCl-C05 | OQ607147 | *Enterococcus avium* | ATCC 14025 | 99.66 | Bacteria | Firmicutes | Bacilli | Lactobacillales | Enterococcaceae | *Enterococcus* |  |
| 127 | PCR02-B29 | DSM479-1%PS-0%NaCl-C01 | OQ599996 | *Terrisporobacter glycolicus* | DSM 1288 | 99.16 | Bacteria | Firmicutes | Clostridia | Clostridiales | Peptostreptococcaceae | *Terrisporobacter* |  |
| 128 | PCR02-B30 | DSM479-1%PS-0%NaCl-C02 | OQ599997 | *Hydrogenoanaerobacterium saccharovorans* | DSM 24774 | 98.82 | Bacteria | Firmicutes | Clostridia | Clostridiales | Oscillospiraceae | *Hydrogenoanaerobacterium* |  |
| 129 | PCR02-B31 | DSM479-1%PS-0%NaCl-C03 | OQ599998 | *Terrisporobacter glycolicus* | DSM 1288 | 99.23 | Bacteria | Firmicutes | Clostridia | Clostridiales | Peptostreptococcaceae | *Terrisporobacter* |  |
| 130 | PCR02-B32 | DSM479-1%PS-0%NaCl-C04 | OQ599999 | *Lacrimispora celerecrescens* | DSM 5628 | 95.72 | Bacteria | Firmicutes | Clostridia | Clostridiales | Lachnospiraceae | *Lacrimispora* |  |
| 131 | PCR02-B33 | DSM479-1%PS-3%NaCl-C01 | OQ600000 | *Clostridioides mangenotii* | DSM 1289 | 99.23 | Bacteria | Firmicutes | Clostridia | Clostridiales | Peptostreptococcaceae | *Clostridioides* |  |
| 132 | PCR02-B34 | DSM479-1%PS-3%NaCl-C02 | OQ600001 | *Clostridioides mangenotii* | DSM 1289 | 99.37 | Bacteria | Firmicutes | Clostridia | Clostridiales | Peptostreptococcaceae | *Clostridioides* |  |
| 133 | PCR02-B35 | DSM479-1%PS-3%NaCl-C03 | OQ600002 | *Clostridioides mangenotii* | DSM 1289 | 99.44 | Bacteria | Firmicutes | Clostridia | Clostridiales | Peptostreptococcaceae | *Clostridioides* |  |
| 134 | PCR02-B36 | DSM479-1%PS-3%NaCl-C04 | OQ600003 | *Clostridioides mangenotii* | DSM 1289 | 93.53 | Bacteria | Firmicutes | Clostridia | Clostridiales | Peptostreptococcaceae | *Clostridioides* |  |
| 135 | PCR02-B37 | DSM479-1%PS-3%NaCl-C05 | OQ600004 | *Clostridioides mangenotii* | DSM 1289 | 99.30 | Bacteria | Firmicutes | Clostridia | Clostridiales | Peptostreptococcaceae | *Clostridioides* |  |
| 136 | PCR02-B38 | DSM479-AFMT-0%NaCl-C01 | OQ600005 | *Oscillibacter valericigenes* | NBRC 101213 | 93.18 | Bacteria | Firmicutes | Clostridia | Clostridiales | Oscillospiraceae | *Oscillibacter* |  |
| 137 | PCR02-B39 | DSM479-AFMT-0%NaCl-C02 | OQ600006 | *Terrisporobacter glycolicus* | DSM 1288 | 99.09 | Bacteria | Firmicutes | Clostridia | Clostridiales | Peptostreptococcaceae | *Terrisporobacter* |  |
| 138 | PCR02-B41 | DSM479-AFMT-0%NaCl-C04 | OQ600007 | *Enterococcus gallinarum* | NBRC 100675 | 99.93 | Bacteria | Firmicutes | Bacilli | Lactobacillales | Enterococcaceae | *Enterococcus* |  |
| 139 | PCR02-B42 | DSM479-AFMT-0%NaCl-C05 | OQ600008 | *Terrisporobacter glycolicus* | DSM 1288 | 99.23 | Bacteria | Firmicutes | Clostridia | Clostridiales | Peptostreptococcaceae | *Terrisporobacter* |  |
| 140 | PCR02-B43 | DSM479-AFMT-0%NaCl-C06 | OQ600009 | *Enterococcus gallinarum* | NBRC 100675 | 99.73 | Bacteria | Firmicutes | Bacilli | Lactobacillales | Enterococcaceae | *Enterococcus* |  |
| 141 | PCR02-B44 | DSM479-AFMT-3%NaCl-C01 | OQ600010 | *Anaerovorax odorimutans* | NorPut1 | 92.78 | Bacteria | Firmicutes | Clostridia | Clostridiales | Mogibacterium_f | *Anaerovorax* |  |
| 142 | PCR02-B45 | DSM479-AFMT-3%NaCl-C02 | OQ600011 | *Enterococcus raffinosus* | NCIMB 12901 | 90.03 | Bacteria | Firmicutes | Bacilli | Lactobacillales | Enterococcaceae | *Enterococcus* |  |
| 143 | PCR02-B46 | DSM479-AFMT-3%NaCl-C03 | OQ600012 | *Terrisporobacter glycolicus* | DSM 1288 | 99.02 | Bacteria | Firmicutes | Clostridia | Clostridiales | Peptostreptococcaceae | *Terrisporobacter* |  |
| 144 | PCR02-B47 | DSM479-AFMT-3%NaCl-C04 | OQ600013 | *Proteus mirabilis* | ATCC 29906 | 99.59 | Bacteria | Proteobacteria | Gammaproteobacteria | Enterobacterales | Morganellaceae | *Proteus* |  |
| 145 | PCR02-B48 | DSM479-AFMT-3%NaCl-C05 | OQ600014 | *Proteus mirabilis* | ATCC 29906 | 99.93 | Bacteria | Proteobacteria | Gammaproteobacteria | Enterobacterales | Morganellaceae | *Proteus* |  |
| 146 | PCR05-B28 | DSM490-1%PS-3%NaCl-C02 | OQ607927 | *Terrisporobacter glycolicus* | DSM 1288 | 99.02 | Bacteria | Firmicutes | Clostridia | Clostridiales | Peptostreptococcaceae | *Terrisporobacter* |  |
| 147 | PCR05-B30 | DSM490-1%PS-3%NaCl-C04 | OQ607928 | *Terrisporobacter glycolicus* | DSM 1288 | 99.16 | Bacteria | Firmicutes | Clostridia | Clostridiales | Peptostreptococcaceae | *Terrisporobacter* |  |
| 148 | PCR05-B32 | DSM490-1%PS-3%NaCl-C06 | OQ607929 | *Enterococcus thailandicus* | DSM 21767 | 98.52 | Bacteria | Firmicutes | Bacilli | Lactobacillales | Enterococcaceae | *Enterococcus* |  |
| 149 | PCR05-B33 | DSM490-1%PS-3%NaCl-C07 | OQ607930 | *Enterococcus thailandicus* | DSM 21767 | 98.38 | Bacteria | Firmicutes | Bacilli | Lactobacillales | Enterococcaceae | *Enterococcus* |  |
| 150 | PCR05-B35 | DSM490-1%PS-3%NaCl-C09 | OQ607931 | *Enterococcus avium* | ATCC 14025 | 99.73 | Bacteria | Firmicutes | Bacilli | Lactobacillales | Enterococcaceae | *Enterococcus* |  |
| 151 | PCR05-B24 | DSM490-AFMT-0%NaCl-C01 | OQ607924 | *Lacrimispora celerecrescens* | DSM 5628 | 99.79 | Bacteria | Firmicutes | Clostridia | Clostridiales | Lachnospiraceae | *Lacrimispora* |  |
| 152 | PCR05-B25 | DSM490-AFMT-0%NaCl-C02 | OQ607925 | *Tessaracoccus aquimaris* | NSG39 | 98.27 | Bacteria | Actinobacteria | Actinomycetia | Propionibacteriales | Propionibacteriaceae | *Tessaracoccus* |  |
| 153 | PCR05-B26 | DSM490-AFMT-0%NaCl-C03 | OQ607926 | *Enterococcus avium* | ATCC 14025 | 99.80 | Bacteria | Firmicutes | Bacilli | Lactobacillales | Enterococcaceae | *Enterococcus* |  |
| 154 | PCR05-B36 | DSM490-AFMT-3%NaCl-C01 | OQ607932 | *Clostridioides mangenotii* | DSM 1289 | 99.37 | Bacteria | Firmicutes | Clostridia | Clostridiales | Peptostreptococcaceae | *Clostridioides* |  |
| 155 | PCR05-B37 | DSM490-AFMT-3%NaCl-C02 | OQ607933 | *Tessaracoccus aquimaris* | NSG39 | 98.20 | Bacteria | Actinobacteria | Actinomycetia | Propionibacteriales | Propionibacteriaceae | *Tessaracoccus* |  |
| 156 | PCR05-B38 | DSM490-AFMT-3%NaCl-C03 | OQ607934 | *Amphibacillus indicireducens* | C40 | 95.03 | Bacteria | Firmicutes | Bacilli | Bacillales | Bacillaceae | *Amphibacillus* |  |
| 157 | PCR05-B40 | DSM490-AFMT-3%NaCl-C05 | OQ607935 | *Tessaracoccus aquimaris* | NSG39 | 98.33 | Bacteria | Actinobacteria | Actinomycetia | Propionibacteriales | Propionibacteriaceae | *Tessaracoccus* |  |
| 158 | PCR05-B41 | DSM490-AFMT-3%NaCl-C06 | OQ607936 | *Clostridioides mangenotii* | DSM 1289 | 99.30 | Bacteria | Firmicutes | Clostridia | Clostridiales | Peptostreptococcaceae | *Clostridioides* |  |
| 159 | PCR05-B42 | DSM490-AFMT-3%NaCl-C07 | OQ607937 | *Urmitella timonensis* | Marseille-P2918 | 94.84 | Bacteria | Firmicutes | Tissierellia | Tissierellales | Tissierellaceae | *Tissierella* |  |
| 160 | PCR05-B43 | DSM490-AFMT-3%NaCl-C08 | OQ607938 | *Clostridium sporogenes* | DSM 795 | 99.86 | Bacteria | Firmicutes | Clostridia | Clostridiales | Clostridiaceae | *Clostridium* |  |
| 161 | PCR06-B01 | DSM684-AFMT-0%NaCl-C01 | OQ608069 | *Hungatella xylanolytica* | X5-1 | 99.31 | Bacteria | Firmicutes | Clostridia | Clostridiales | Lachnospiraceae | *Lacrimispora* |  |
| 162 | PCR06-B02 | DSM684-AFMT-0%NaCl-C02 | OQ608070 | *Enterococcus hulanensis* | 190-7 | 99.79 | Bacteria | Firmicutes | Bacilli | Lactobacillales | Enterococcaceae | *Enterococcus* |  |
| 163 | PCR06-B03 | DSM684-AFMT-0%NaCl-C03 | OQ608071 | *Enterococcus avium* | ATCC 14025 | 99.80 | Bacteria | Firmicutes | Bacilli | Lactobacillales | Enterococcaceae | *Enterococcus* |  |
| 164 | PCR06-B05 | DSM684-AFMT-0%NaCl-C05 | OQ608072 | *Obesumbacterium proteus* | DSM 2777 | 99.52 | Bacteria | Proteobacteria | Gammaproteobacteria | Enterobacterales | Hafniaceae | *Hafnia* |  |
| 165 | PCR06-B08 | DSM684-AFMT-0%NaCl-C08 | OQ608073 | *Enterococcus avium* | ATCC 14025 | 99.80 | Bacteria | Firmicutes | Bacilli | Lactobacillales | Enterococcaceae | *Enterococcus* |  |
| 166 | PCR06-B09 | DSM684-AFMT-0%NaCl-C09 | OQ608074 | *Obesumbacterium proteus* | DSM 2777 | 99.39 | Bacteria | Proteobacteria | Gammaproteobacteria | Enterobacterales | Hafniaceae | *Hafnia* |  |
| 167 | PCR06-B10 | DSM684-AFMT-0%NaCl-C10 | OQ608075 | *Herbaspirillum huttiense subsp. huttiense* | ATCC 14670 | 99.93 | Bacteria | Proteobacteria | Betaproteobacteria | Burkholderiales | Oxalobacteraceae | *Herbaspirillum* | *Herbaspirillum huttiense* |
| 168 | PCR03-B30 | DSM713-1%PS-0%NaCl-C01 | OQ607153 | *Enterococcus faecalis* | ATCC 19433 | 99.87 | Bacteria | Firmicutes | Bacilli | Lactobacillales | Enterococcaceae | *Enterococcus* |  |
| 169 | PCR03-B31 | DSM713-1%PS-0%NaCl-C02 | OQ607154 | *Enterococcus faecalis* | ATCC 19433 | 99.73 | Bacteria | Firmicutes | Bacilli | Lactobacillales | Enterococcaceae | *Enterococcus* |  |
| 170 | PCR03-B32 | DSM713-1%PS-0%NaCl-C03 | OQ607155 | *Enterococcus faecalis* | ATCC 19433 | 99.93 | Bacteria | Firmicutes | Bacilli | Lactobacillales | Enterococcaceae | *Enterococcus* |  |
| 171 | PCR03-B33 | DSM713-1%PS-0%NaCl-C04 | OQ607156 | *Enterococcus faecalis* | ATCC 19433 | 99.93 | Bacteria | Firmicutes | Bacilli | Lactobacillales | Enterococcaceae | *Enterococcus* |  |
| 172 | PCR03-B34 | DSM713-1%PS-0%NaCl-C05 | OQ607157 | *Enterococcus faecalis* | ATCC 19433 | 100.00 | Bacteria | Firmicutes | Bacilli | Lactobacillales | Enterococcaceae | *Enterococcus* |  |
| 173 | PCR03-B35 | DSM713-1%PS-3%NaCl-C01 | OQ607158 | *Enterococcus faecalis* | ATCC 19433 | 99.80 | Bacteria | Firmicutes | Bacilli | Lactobacillales | Enterococcaceae | *Enterococcus* |  |
| 174 | PCR03-B36 | DSM713-1%PS-3%NaCl-C02 | OQ607159 | *Enterococcus faecalis* | ATCC 19433 | 100.00 | Bacteria | Firmicutes | Bacilli | Lactobacillales | Enterococcaceae | *Enterococcus* |  |
| 175 | PCR03-B37 | DSM713-1%PS-3%NaCl-C03 | OQ607160 | *Enterococcus faecalis* | ATCC 19433 | 99.93 | Bacteria | Firmicutes | Bacilli | Lactobacillales | Enterococcaceae | *Enterococcus* |  |
| 176 | PCR03-B38 | DSM713-1%PS-3%NaCl-C04 | OQ607161 | *Enterococcus faecalis* | ATCC 19433 | 99.93 | Bacteria | Firmicutes | Bacilli | Lactobacillales | Enterococcaceae | *Enterococcus* |  |
| 177 | PCR03-B40 | DSM713-AFMT-0%NaCl-C02 | OQ607162 | *Lacrimispora celerecrescens* | DSM 5628 | 99.72 | Bacteria | Firmicutes | Clostridia | Clostridiales | Lachnospiraceae | *Lacrimispora* |  |
| 178 | PCR03-B41 | DSM713-AFMT-0%NaCl-C03 | OQ607163 | *Enterococcus thailandicus* | DSM 21767 | 99.87 | Bacteria | Firmicutes | Bacilli | Lactobacillales | Enterococcaceae | *Enterococcus* |  |
| 179 | PCR03-B42 | DSM713-AFMT-0%NaCl-C04 | OQ607164 | *Lacrimispora celerecrescens* | DSM 5628 | 99.72 | Bacteria | Firmicutes | Clostridia | Clostridiales | Lachnospiraceae | *Lacrimispora* |  |
| 180 | PCR03-B43 | DSM713-AFMT-0%NaCl-C05 | OQ607165 | *Lacrimispora celerecrescens* | DSM 5628 | 99.72 | Bacteria | Firmicutes | Clostridia | Clostridiales | Lachnospiraceae | *Lacrimispora* |  |
| 181 | PCR03-B44 | DSM713-AFMT-3%NaCl-C01 | OQ607166 | *Enterococcus faecalis* | ATCC 19433 | 99.93 | Bacteria | Firmicutes | Bacilli | Lactobacillales | Enterococcaceae | *Enterococcus* |  |
| 182 | PCR03-B45 | DSM713-AFMT-3%NaCl-C02 | OQ607167 | *Enterococcus faecalis* | ATCC 19433 | 99.93 | Bacteria | Firmicutes | Bacilli | Lactobacillales | Enterococcaceae | *Enterococcus* |  |
| 183 | PCR03-B46 | DSM713-AFMT-3%NaCl-C03 | OQ607168 | *Enterococcus faecalis* | ATCC 19433 | 99.87 | Bacteria | Firmicutes | Bacilli | Lactobacillales | Enterococcaceae | *Enterococcus* |  |
| 184 | PCR03-B47 | DSM713-AFMT-3%NaCl-C04 | OQ607169 | *Enterococcus faecalis* | ATCC 19433 | 99.93 | Bacteria | Firmicutes | Bacilli | Lactobacillales | Enterococcaceae | *Enterococcus* |  |
| 185 | PCR03-B48 | DSM713-AFMT-3%NaCl-C05 | OQ607170 | *Enterococcus faecalis* | ATCC 19433 | 100.00 | Bacteria | Firmicutes | Bacilli | Lactobacillales | Enterococcaceae | *Enterococcus* |  |
| 186 | PCR04-B25 | DSM905-1%PS-0%NaCl-C03 | OQ607782 | *Enterococcus gallinarum* | NBRC 100675 | 99.87 | Bacteria | Firmicutes | Bacilli | Lactobacillales | Enterococcaceae | *Enterococcus* |  |
| 187 | PCR04-B28 | DSM905-1%PS-3%NaCl-C01 | OQ607783 | *Enterococcus gallinarum* | NBRC 100675 | 99.73 | Bacteria | Firmicutes | Bacilli | Lactobacillales | Enterococcaceae | *Enterococcus* |  |
| 188 | PCR04-B29 | DSM905-1%PS-3%NaCl-C02 | OQ607784 | *Enterococcus gallinarum* | NBRC 100675 | 99.66 | Bacteria | Firmicutes | Bacilli | Lactobacillales | Enterococcaceae | *Enterococcus* |  |
| 189 | PCR04-B30 | DSM905-1%PS-3%NaCl-C03 | OQ607785 | *Enterococcus gallinarum* | NBRC 100675 | 99.93 | Bacteria | Firmicutes | Bacilli | Lactobacillales | Enterococcaceae | *Enterococcus* |  |
| 190 | PCR04-B32 | DSM905-1%PS-3%NaCl-C05 | OQ607786 | *Enterococcus phoeniculicola* | ATCC BAA-412 | 99.80 | Bacteria | Firmicutes | Bacilli | Lactobacillales | Enterococcaceae | *Enterococcus* |  |
| 191 | PCR04-B35 | DSM905-AFMT-0%NaCl-C03 | OQ607787 | *Burkholderia contaminans* | LMG 23361 | 99.93 | Bacteria | Proteobacteria | Betaproteobacteria | Burkholderiales | Burkholderiaceae | *Burkholderia* |  |
| 192 | PCR04-B38 | DSM905-AFMT-3%NaCl-C01 | OQ607788 | *Morganella morganii subsp. sibonii* | DSM 14850 | 99.48 | Bacteria | Proteobacteria | Gammaproteobacteria | Enterobacterales | Morganellaceae | *Morganella* | *Morganella morganii* |
| 193 | PCR04-B39 | DSM905-AFMT-3%NaCl-C02 | OQ607789 | *Morganella morganii subsp. sibonii* | DSM 14850 | 99.48 | Bacteria | Proteobacteria | Gammaproteobacteria | Enterobacterales | Morganellaceae | *Morganella* | *Morganella morganii* |
| 194 | PCR04-B40 | DSM905-AFMT-3%NaCl-C03 | OQ607790 | *Morganella morganii subsp. sibonii* | DSM 14850 | 99.63 | Bacteria | Proteobacteria | Gammaproteobacteria | Enterobacterales | Morganellaceae | *Morganella* | *Morganella morganii* |
| 195 | PCR02-B01 | DSM924-AFMT-0%NaCl-C01 | OQ599969 | *Clostridium argentinense* | ATCC 27322 | 98.53 | Bacteria | Firmicutes | Clostridia | Clostridiales | Clostridiaceae | *Clostridium* |  |
| 196 | PCR02-B02 | DSM924-AFMT-0%NaCl-C02 | OQ599970 | *Clostridium argentinense* | ATCC 27322 | 99.02 | Bacteria | Firmicutes | Clostridia | Clostridiales | Clostridiaceae | *Clostridium* |  |
| 197 | PCR02-B03 | DSM924-AFMT-0%NaCl-C03 | OQ599971 | *Clostridium argentinense* | ATCC 27322 | 97.63 | Bacteria | Firmicutes | Clostridia | Clostridiales | Clostridiaceae | *Clostridium* |  |
| 198 | PCR02-B04 | DSM924-AFMT-0%NaCl-C04 | OQ599972 | *Clostridioides mangenotii* | DSM 1289 | 99.44 | Bacteria | Firmicutes | Clostridia | Clostridiales | Peptostreptococcaceae | *Clostridioides* |  |
| 199 | PCR02-B05 | DSM924-AFMT-0%NaCl-C05 | OQ599973 | *Lacrimispora sphenoides* | JCM 1415 | 92.04 | Bacteria | Firmicutes | Clostridia | Clostridiales | Lachnospiraceae | *Lacrimispora* |  |
| 200 | PCR02-B06 | DSM924-AFMT-0%NaCl-C06 | OQ599974 | *Clostridium argentinense* | ATCC 27322 | 99.09 | Bacteria | Firmicutes | Clostridia | Clostridiales | Clostridiaceae | *Clostridium* |  |
| 201 | PCR02-B07 | DSM924-AFMT-0%NaCl-C07 | OQ599975 | *Clostridium argentinense* | ATCC 27322 | 99.09 | Bacteria | Firmicutes | Clostridia | Clostridiales | Clostridiaceae | *Clostridium* |  |
| 202 | PCR02-B08 | DSM924-AFMT-0.5%NaCl-C01 | OQ599976 | *Enterococcus avium* | ATCC 14025 | 99.86 | Bacteria | Firmicutes | Bacilli | Lactobacillales | Enterococcaceae | *Enterococcus* |  |
| 203 | PCR02-B09 | DSM924-AFMT-0.5%NaCl-C02 | OQ599977 | *Tissierella pigra* | WCA3-693-APC-4 | 99.59 | Bacteria | Firmicutes | Tissierellia | Tissierellales | Tissierellaceae | *Tissierella* |  |
| 204 | PCR02-B10 | DSM924-AFMT-0.5%NaCl-C03 | OQ599978 | *Enterococcus avium* | ATCC 14025 | 99.93 | Bacteria | Firmicutes | Bacilli | Lactobacillales | Enterococcaceae | *Enterococcus* |  |
| 205 | PCR02-B11 | DSM924-AFMT-0.5%NaCl-C04 | OQ599979 | *Tissierella pigra* | WCA3-693-APC-4 | 99.52 | Bacteria | Firmicutes | Tissierellia | Tissierellales | Tissierellaceae | *Tissierella* |  |
| 206 | PCR02-B12 | DSM924-AFMT-0.5%NaCl-C05 | OQ599980 | *Tissierella pigra* | WCA3-693-APC-4 | 99.52 | Bacteria | Firmicutes | Tissierellia | Tissierellales | Tissierellaceae | *Tissierella* |  |
| 207 | PCR02-B14 | DSM924-AFMT-3%NaCl-C01 | OQ599981 | *Morganella morganii subsp. morganii* | ATCC 25830 | 98.43 | Bacteria | Proteobacteria | Gammaproteobacteria | Enterobacterales | Morganellaceae | *Morganella* | *Morganella morganii* |
| 208 | PCR02-B15 | DSM924-AFMT-3%NaCl-C02 | OQ599982 | *Enterococcus faecalis* | ATCC 19433 | 99.87 | Bacteria | Firmicutes | Bacilli | Lactobacillales | Enterococcaceae | *Enterococcus* |  |
| 209 | PCR02-B16 | DSM924-AFMT-3%NaCl-C03 | OQ599983 | *Enterococcus hulanensis* | 190-7 | 99.93 | Bacteria | Firmicutes | Bacilli | Lactobacillales | Enterococcaceae | *Enterococcus* |  |
| 210 | PCR02-B17 | DSM924-AFMT-3%NaCl-C04 | OQ599984 | *Enterococcus gallinarum* | NBRC 100675 | 99.60 | Bacteria | Firmicutes | Bacilli | Lactobacillales | Enterococcaceae | *Enterococcus* |  |
| 211 | PCR02-B18 | DSM924-AFMT-3%NaCl-C05 | OQ599985 | *Enterococcus avium* | ATCC 14025 | 99.66 | Bacteria | Firmicutes | Bacilli | Lactobacillales | Enterococcaceae | *Enterococcus* |  |
| 212 | PCR02-B19 | DSM924-AFMT-3%NaCl-C06 | OQ599986 | *Enterococcus faecalis* | ATCC 19433 | 99.80 | Bacteria | Firmicutes | Bacilli | Lactobacillales | Enterococcaceae | *Enterococcus* |  |
| 213 | PCR02-B20 | DSM924-AFMT-3%NaCl-C07 | OQ599987 | *Clostridioides mangenotii* | DSM 1289 | 98.95 | Bacteria | Firmicutes | Clostridia | Clostridiales | Peptostreptococcaceae | *Clostridioides* |  |
| 214 | PCR02-B21 | DSM924-AFMT-3%NaCl-C08 | OQ599988 | *Enterococcus gallinarum* | NBRC 100675 | 99.73 | Bacteria | Firmicutes | Bacilli | Lactobacillales | Enterococcaceae | *Enterococcus* |  |
| 215 | PCR02-B22 | DSM924-AFMT-3%NaCl-C09 | OQ599989 | *Clostridioides mangenotii* | DSM 1289 | 99.02 | Bacteria | Firmicutes | Clostridia | Clostridiales | Peptostreptococcaceae | *Clostridioides* |  |
| 216 | PCR02-B23 | DSM924-AFMT-3%NaCl-C10 | OQ599990 | *Enterococcus gallinarum* | NBRC 100675 | 99.87 | Bacteria | Firmicutes | Bacilli | Lactobacillales | Enterococcaceae | *Enterococcus* |  |
| 217 | PCR02-B24 | DSM924-AFMT-3%NaCl-C12 | OQ599991 | *Clostridioides mangenotii* | DSM 1289 | 99.30 | Bacteria | Firmicutes | Clostridia | Clostridiales | Peptostreptococcaceae | *Clostridioides* |  |
| 218 | PCR02-B25 | DSM924-AFMT-3%NaCl-C13 | OQ599992 | *Enterococcus gallinarum* | NBRC 100675 | 99.87 | Bacteria | Firmicutes | Bacilli | Lactobacillales | Enterococcaceae | *Enterococcus* |  |
| 219 | PCR02-B26 | DSM924-AFMT-3%NaCl-C14 | OQ599993 | *Enterococcus faecalis* | ATCC 19433 | 99.93 | Bacteria | Firmicutes | Bacilli | Lactobacillales | Enterococcaceae | *Enterococcus* |  |
| 220 | PCR02-B27 | DSM924-AFMT-3%NaCl-C15 | OQ599994 | *Enterococcus faecalis* | ATCC 19433 | 99.93 | Bacteria | Firmicutes | Bacilli | Lactobacillales | Enterococcaceae | *Enterococcus* |  |
| 221 | PCR02-B28 | DSM924-AFMT-3%NaCl-C16 | OQ599995 | *Enterococcus faecalis* | ATCC 19433 | 100.00 | Bacteria | Firmicutes | Bacilli | Lactobacillales | Enterococcaceae | *Enterococcus* |  |
| 222 | PCR06-B12 | DSM960-1%PS-0%NaCl-C02 | OQ608076 | *Morganella morganii subsp. morganii* | ATCC 25830 | 99.25 | Bacteria | Proteobacteria | Gammaproteobacteria | Enterobacterales | Morganellaceae | *Morganella* | *Morganella morganii* |
| 223 | PCR06-B13 | DSM960-1%PS-0%NaCl-C03 | OQ608077 | *Herbaspirillum huttiense subsp. putei* | IAM 15032 | 100.00 | Bacteria | Proteobacteria | Betaproteobacteria | Burkholderiales | Oxalobacteraceae | *Herbaspirillum* | *Herbaspirillum huttiense* |
| 224 | PCR06-B15 | DSM960-1%PS-3%NaCl-C01 | OQ608078 | *Proteus mirabilis* | ATCC 29906 | 99.86 | Bacteria | Proteobacteria | Gammaproteobacteria | Enterobacterales | Morganellaceae | *Proteus* |  |
| 225 | PCR06-B18 | DSM960-AFMT-0%NaCl-C01 | OQ608079 | *Shigella flexneri* | ATCC 29903 | 99.66 | Bacteria | Proteobacteria | Gammaproteobacteria | Enterobacterales | Enterobacteriaceae | *Escherichia* |  |
| 226 | PCR06-B23 | DSM960-AFMT-0%NaCl-C03 | OQ608080 | *Clostridium malenominatum* | DSM 1127 | 98.33 | Bacteria | Firmicutes | Clostridia | Clostridiales | Clostridiaceae | *Clostridium* |  |


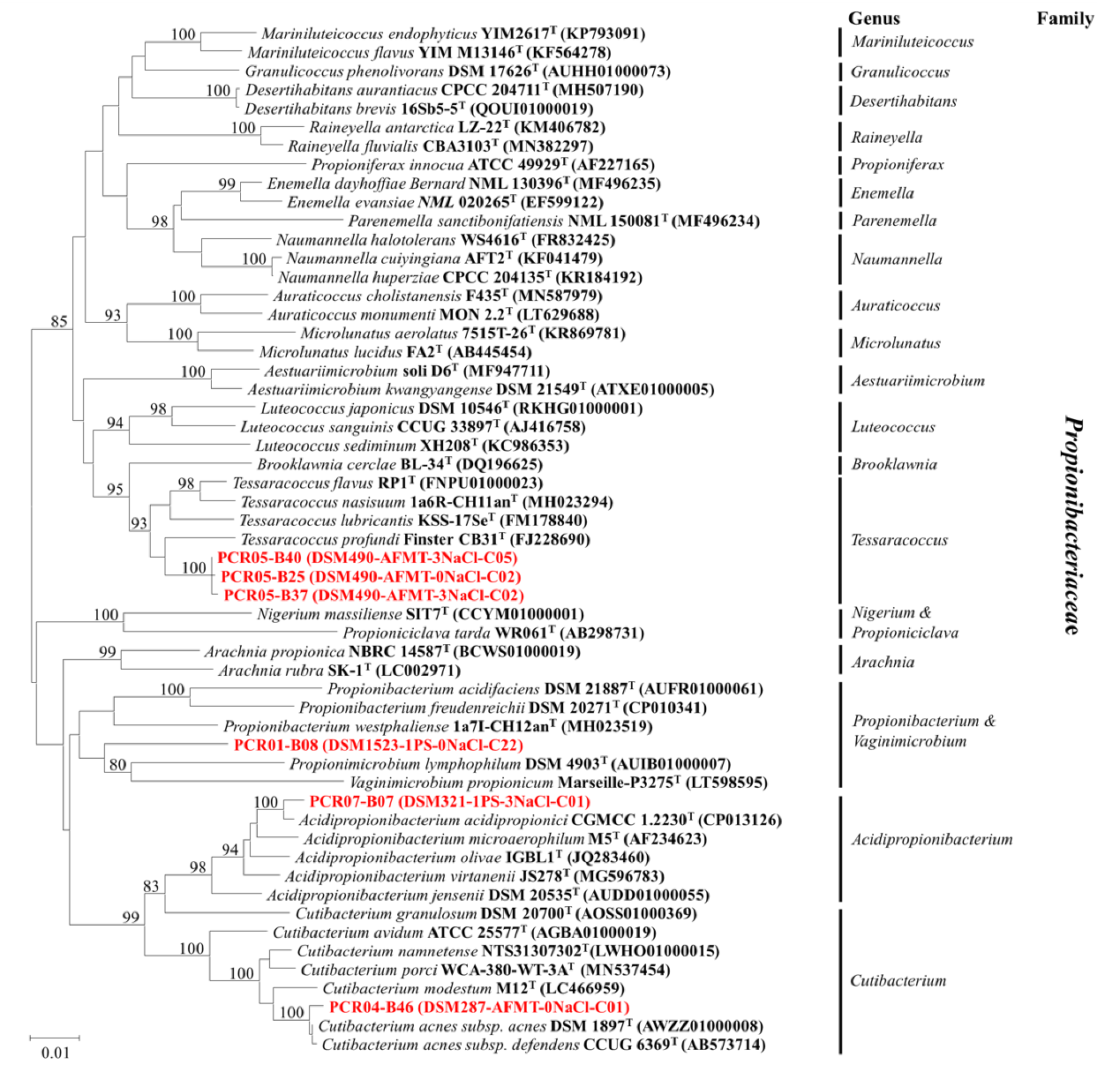


**Figure S1 |** Neighbor-joining phylogenetic tree of culturable anaerobic bacteria isolated from the Tenebrio molito gut belonging to Propionibacteriaceae based on 16S rRNA gene sequences and the effective sequence length was ~1450 bp


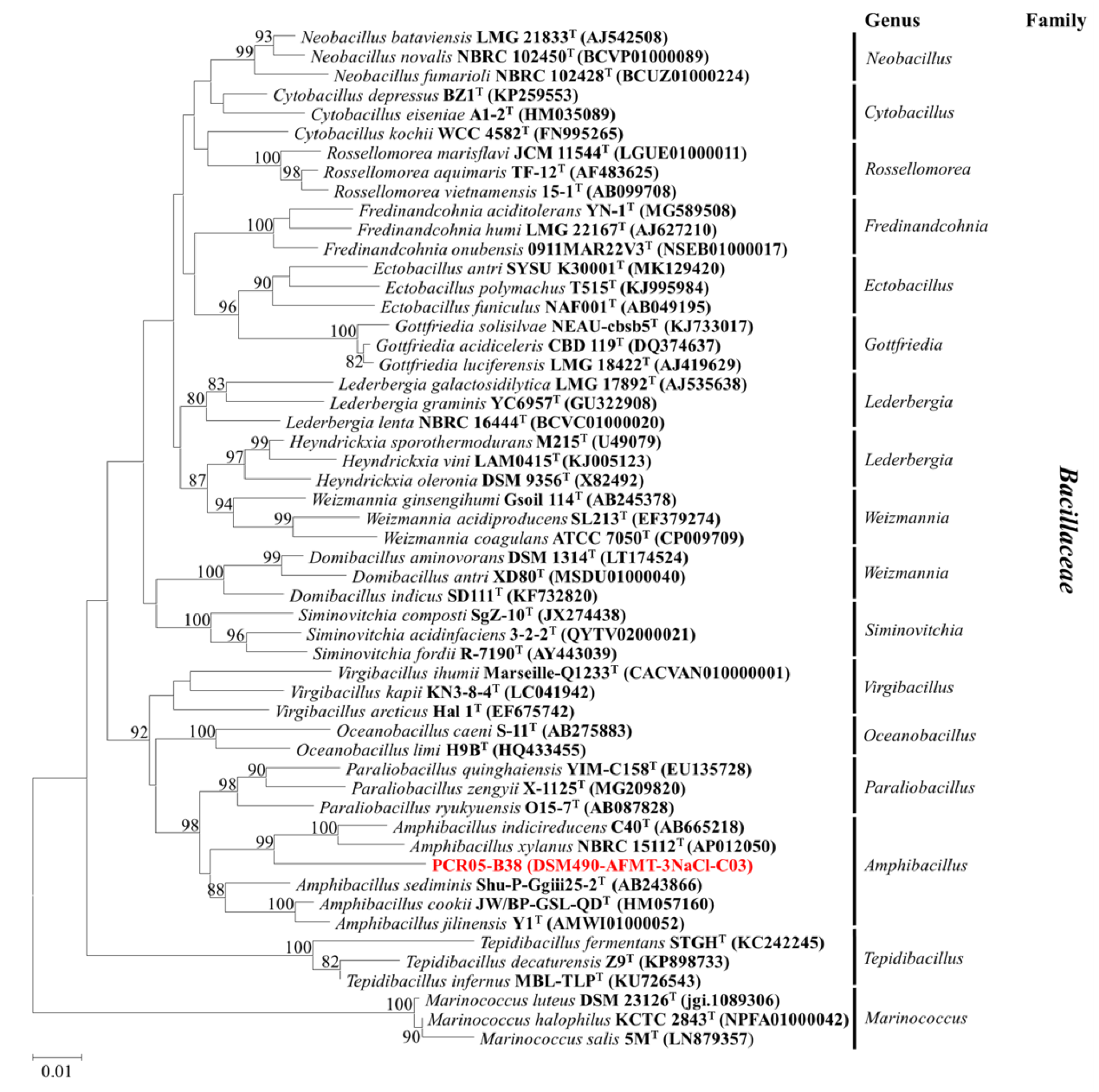


**Figure S2 |** Neighbor-joining phylogenetic tree of culturable anaerobic bacteria isolated from the *Tenebrio molito* gut belonging to *Bacillaceae* based on 16S rRNA gene sequences and the effective sequence length was ~1450 bp


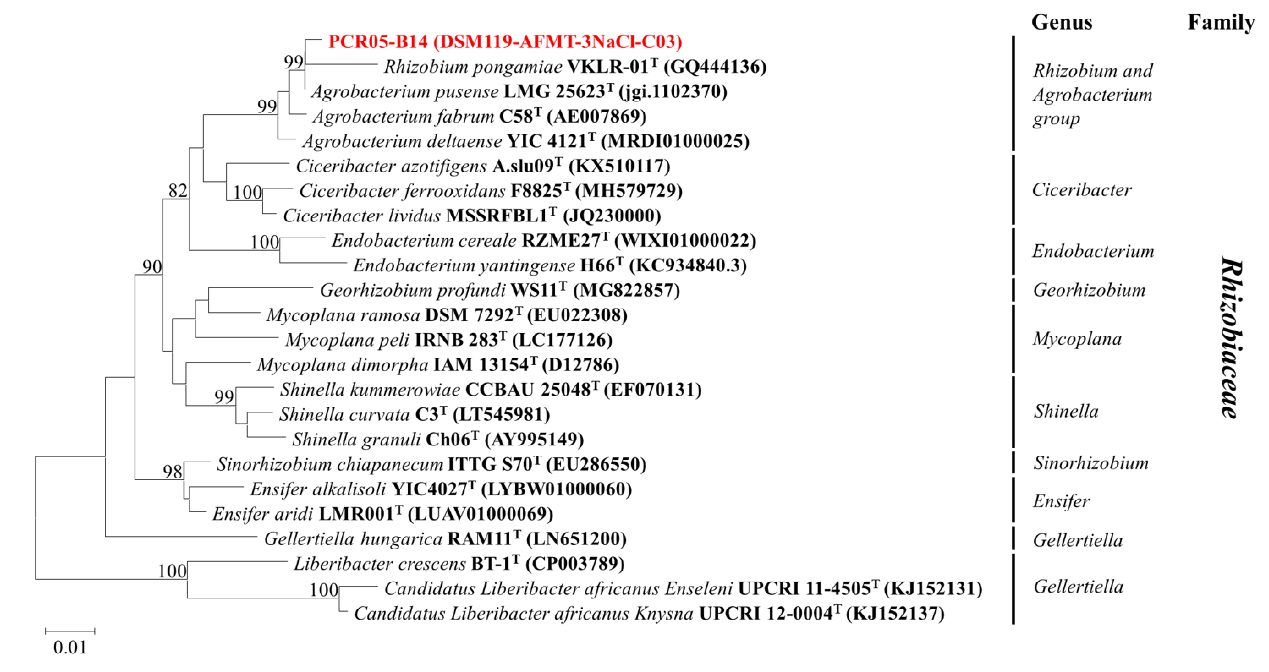


**Figure S3 |** Neighbor-joining phylogenetic tree of culturable anaerobic bacteria isolated from the *Tenebrio molito* gut belonging to *Rhizobiaceae* based on 16S rRNA gene sequences and the effective sequence length was ~1450 bp


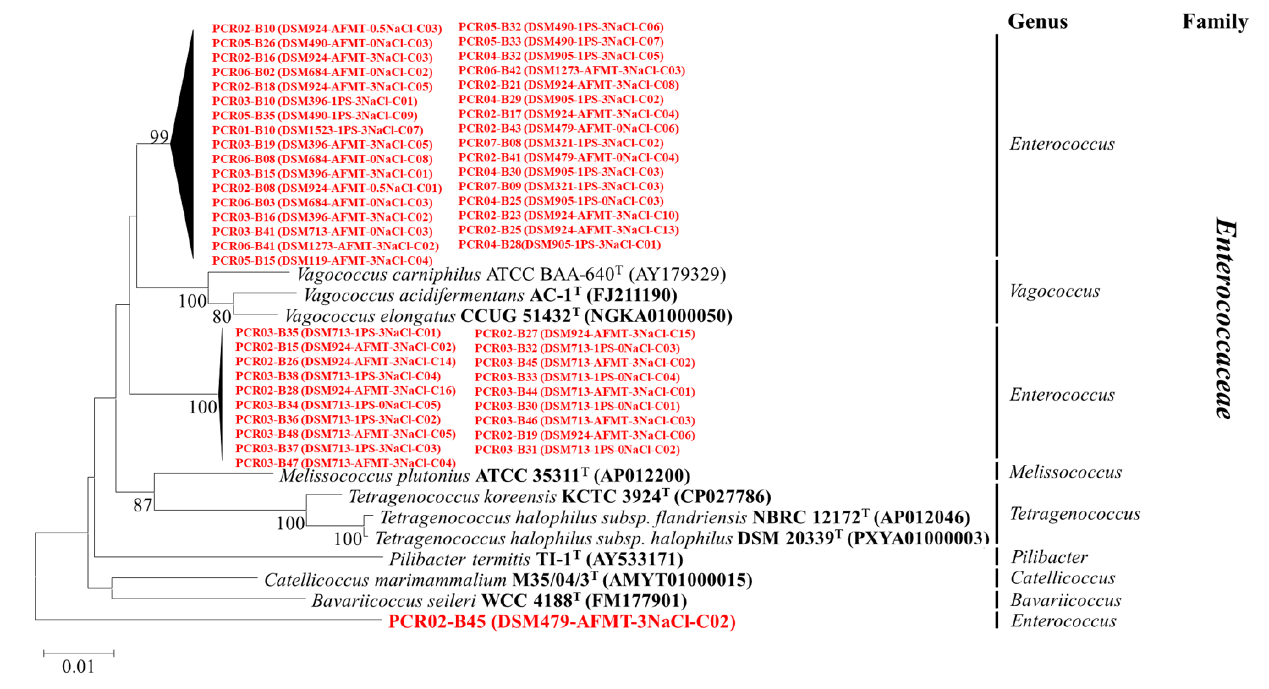


**Figure S4 |** Neighbor-joining phylogenetic tree of culturable anaerobic bacteria isolated from the *Tenebrio molito* gut belonging to *Enterococcaceae* based on 16S rRNA gene sequences and the effective sequence length was ~1450 bp


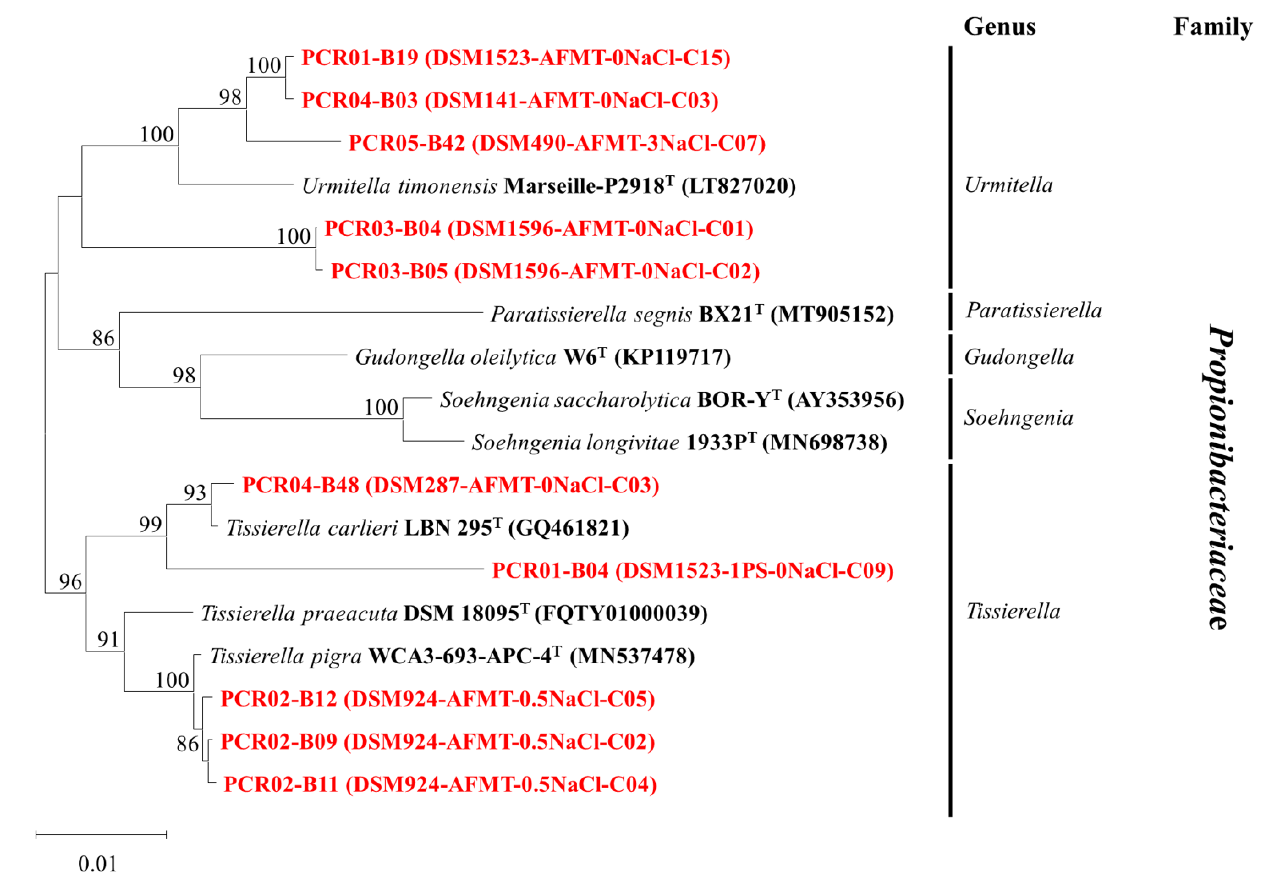


**Figure S5 |** Neighbor-joining phylogenetic tree of culturable anaerobic bacteria isolated from the *Tenebrio molito* gut belonging to *Propionibacteriaceae* based on 16S rRNA gene sequences and the effective sequence length was ~1450 bp


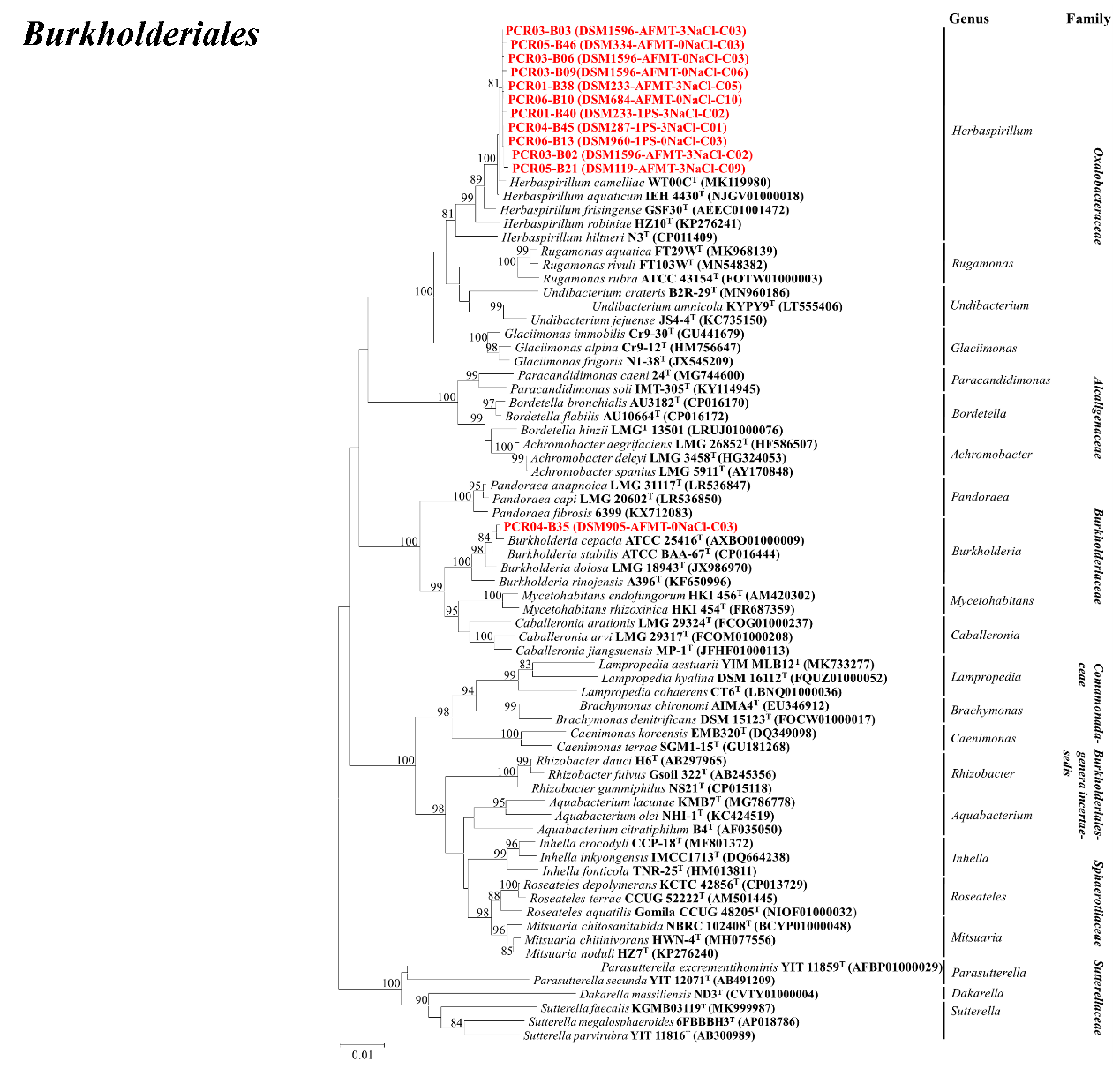


**Figure S6 |** Neighbor-joining phylogenetic tree of culturable anaerobic bacteria isolated from the *Tenebrio molito* gut belonging to *Bukholderiales* based on 16S rRNA gene sequences and the effective sequence length was ~1450 bp


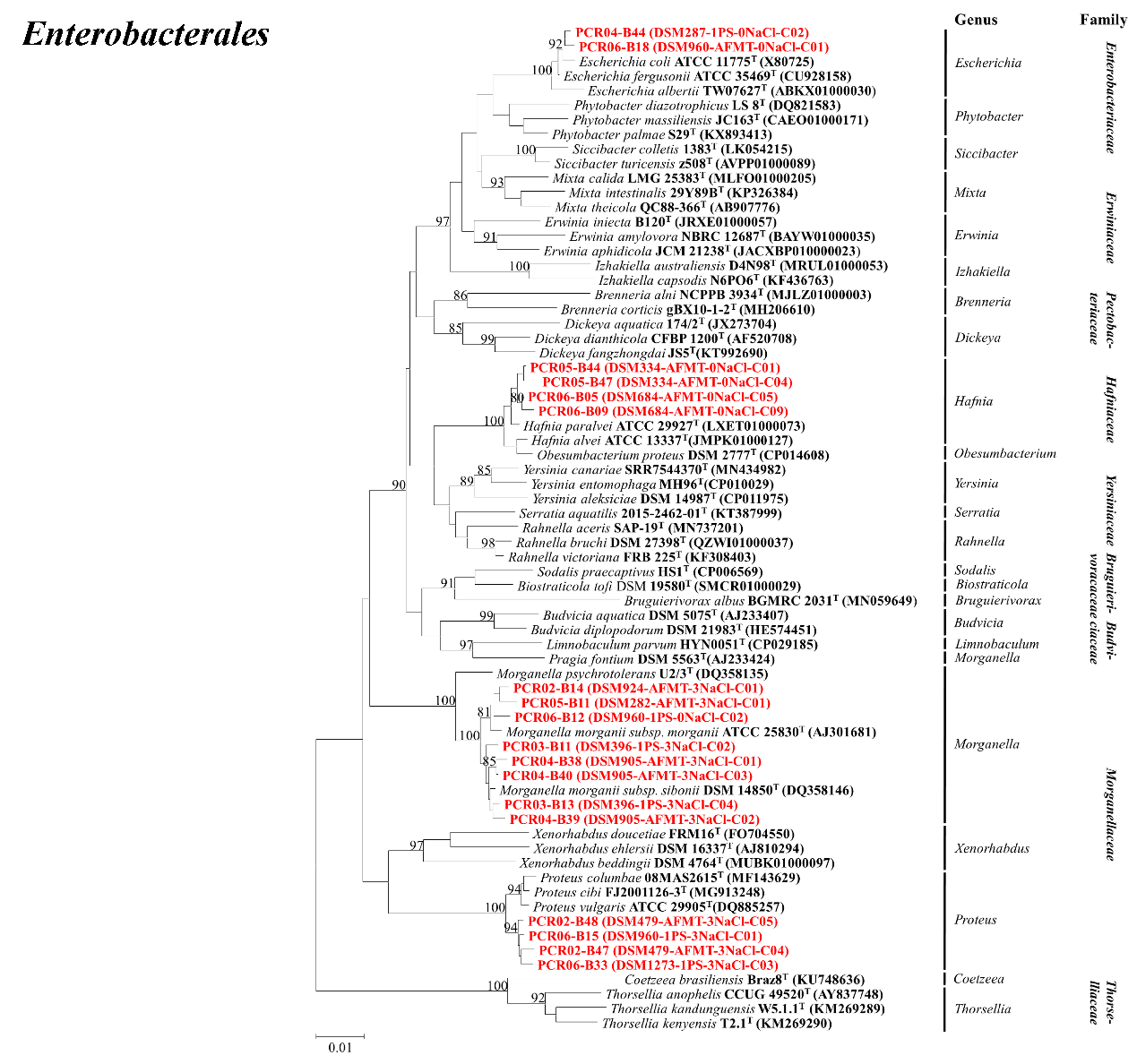


**Figure S7 |** Neighbor-joining phylogenetic tree of culturable anaerobic bacteria isolated from the *Tenebrio molito* gut belonging to *Enterobacterales* based on 16S rRNA gene sequences and the effective sequence length was ~1450 bp


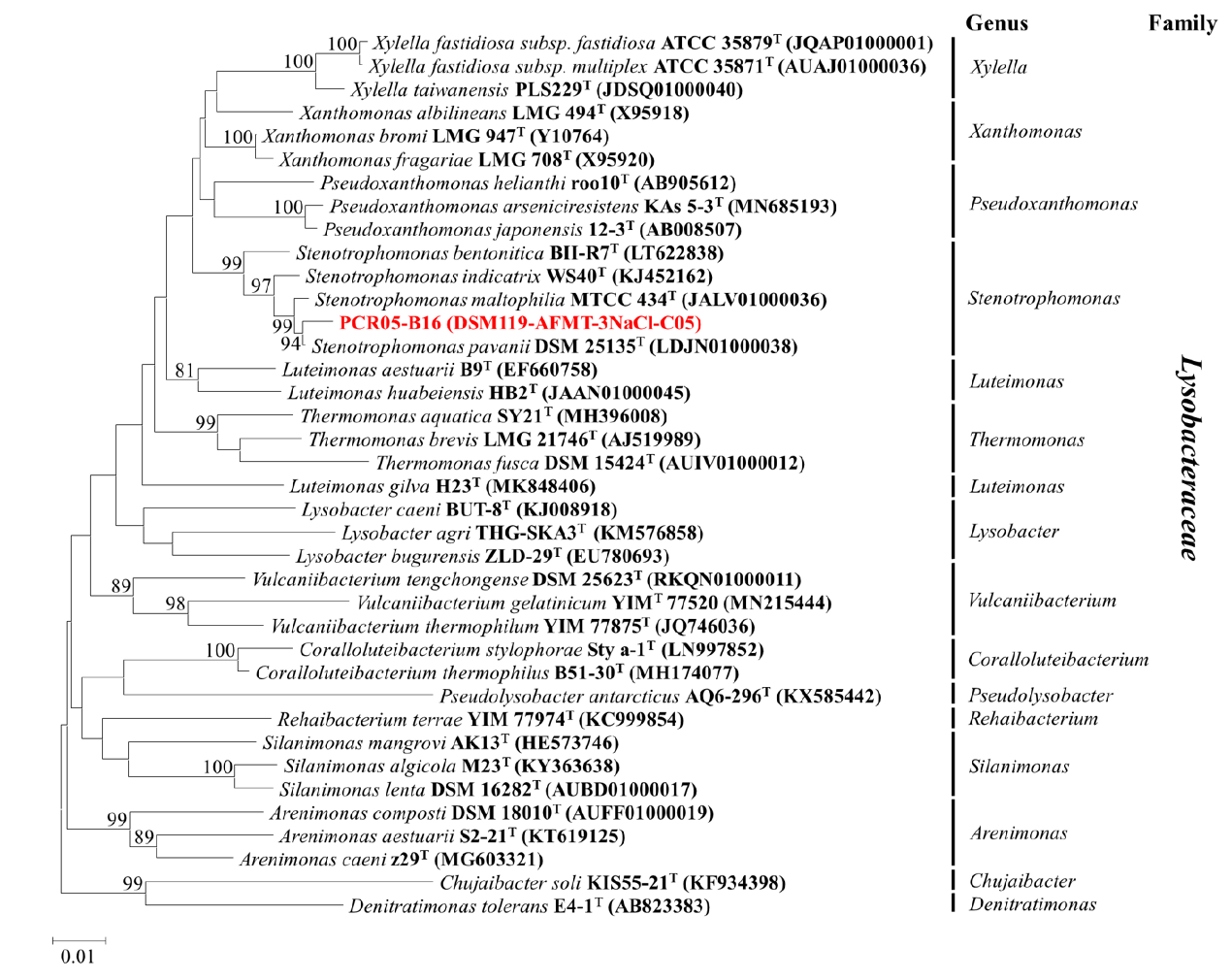


**Figure S8 |** Neighbor-joining phylogenetic tree of culturable anaerobic bacteria isolated from the *Tenebrio molito* gut belonging to *Lysobacteraceae* based on 16S rRNA gene sequences and the effective sequence length was ~1450 bp


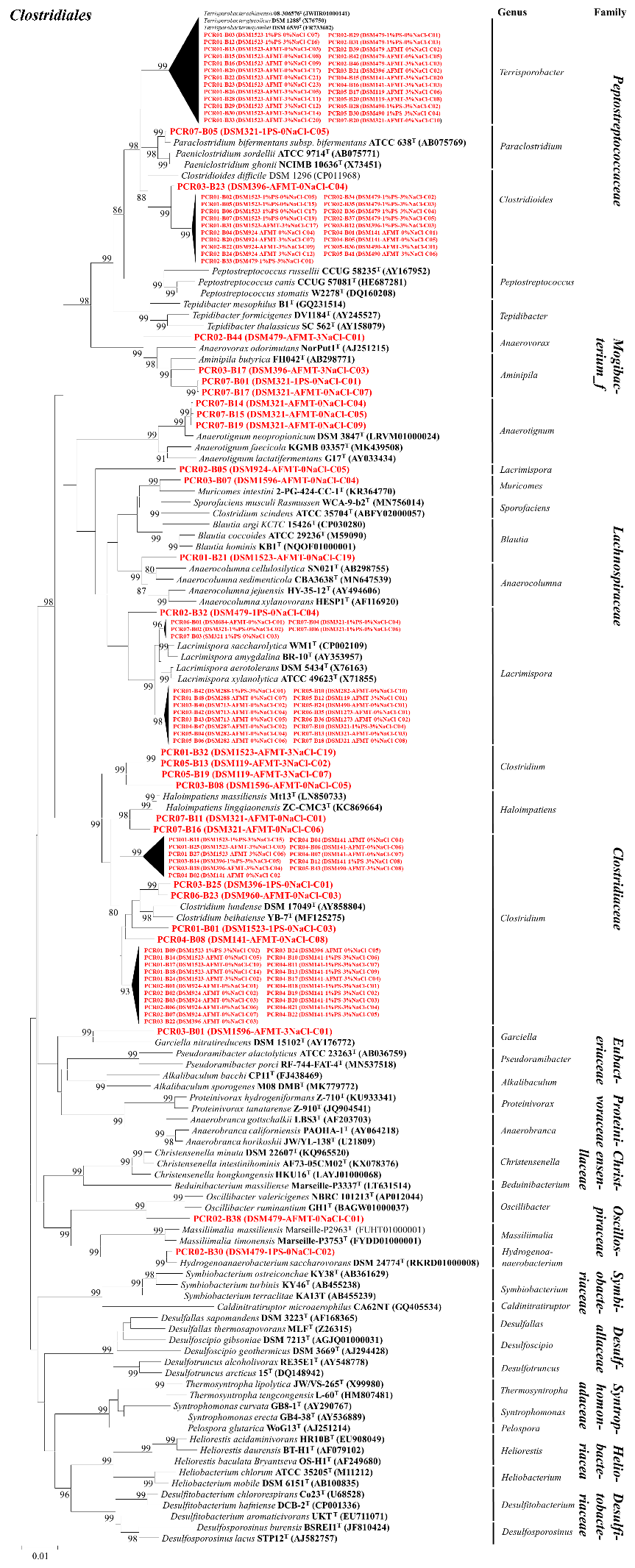


**Figure S9 |** Neighbor-joining phylogenetic tree of culturable anaerobic bacteria isolated from the *Tenebrio molito* gut belonging to *Clostridiales* based on 16S rRNA gene sequences and the effective sequence length was ~1450 bp
